# Rapid Transfer of Plant Photosynthates to Soil Bacteria via Ectomycorrhizal Hyphae and Its Interaction With Nitrogen Availability

**DOI:** 10.3389/fmicb.2019.00168

**Published:** 2019-02-26

**Authors:** Stefan Gorka, Marlies Dietrich, Werner Mayerhofer, Raphael Gabriel, Julia Wiesenbauer, Victoria Martin, Qing Zheng, Bruna Imai, Judith Prommer, Marieluise Weidinger, Peter Schweiger, Stephanie A. Eichorst, Michael Wagner, Andreas Richter, Arno Schintlmeister, Dagmar Woebken, Christina Kaiser

**Affiliations:** ^1^Department of Microbiology and Ecosystem Science, Research Network “Chemistry meets Microbiology”, University of Vienna, Vienna, Austria; ^2^Core Facility Cell Imaging and Ultrastructure Research, University of Vienna, Vienna, Austria; ^3^Large-Instrument Facility for Advanced Isotope Research, University of Vienna, Vienna, Austria

**Keywords:** ectomycorrhiza, hyphal carbon transfer, hyphosphere bacteria, mycorrhizosphere, hyphosphere priming, PLFAs, NanoSIMS

## Abstract

Plant roots release recent photosynthates into the rhizosphere, accelerating decomposition of organic matter by saprotrophic soil microbes (“rhizosphere priming effect”) which consequently increases nutrient availability for plants. However, about 90% of all higher plant species are mycorrhizal, transferring a significant fraction of their photosynthates directly to their fungal partners. Whether mycorrhizal fungi pass on plant-derived carbon (C) to bacteria in root-distant soil areas, i.e., incite a “hyphosphere priming effect,” is not known. Experimental evidence for C transfer from mycorrhizal hyphae to soil bacteria is limited, especially for ectomycorrhizal systems. As ectomycorrhizal fungi possess enzymatic capabilities to degrade organic matter themselves, it remains unclear whether they cooperate with soil bacteria by providing photosynthates, or compete for available nutrients. To investigate a possible C transfer from ectomycorrhizal hyphae to soil bacteria, and its response to changing nutrient availability, we planted young beech trees (*Fagus sylvatica*) into “split-root” boxes, dividing their root systems into two disconnected soil compartments. Each of these compartments was separated from a litter compartment by a mesh penetrable for fungal hyphae, but not for roots. Plants were exposed to a ^13^C-CO_2_-labeled atmosphere, while ^15^N-labeled ammonium and amino acids were added to one side of the split-root system. We found a rapid transfer of recent photosynthates via ectomycorrhizal hyphae to bacteria in root-distant soil areas. Fungal and bacterial phospholipid fatty acid (PLFA) biomarkers were significantly enriched in hyphae-exclusive compartments 24 h after ^13^C-CO_2_-labeling. Isotope imaging with nanometer-scale secondary ion mass spectrometry (NanoSIMS) allowed for the first time *in situ* visualization of plant-derived C and N taken up by an extraradical fungal hypha, and in microbial cells thriving on hyphal surfaces. When N was added to the litter compartments, bacterial biomass, and the amount of incorporated ^13^C strongly declined. Interestingly, this effect was also observed in adjacent soil compartments where added N was only available for bacteria through hyphal transport, indicating that ectomycorrhizal fungi were acting on soil bacteria. Together, our results demonstrate that (i) ectomycorrhizal hyphae rapidly transfer plant-derived C to bacterial communities in root-distant areas, and (ii) this transfer promptly responds to changing soil nutrient conditions.

## Introduction

Plants allocate considerable amounts of recently photoassimilated carbon (C) to their belowground biomass. A substantial fraction of this C is further transferred into the adjacent soil, either via direct root exudation (Walker et al., 2003; Dilkes et al., 2004; Bahn et al., 2009), or mycorrhizal fungi (Leake et al., 2001; Johnson et al., 2002; Simard et al., 2003). Root exudation is thought to trigger *rhizosphere priming*, that is, the acceleration of microbial soil organic matter (SOM) decomposition by increased availability of labile microbial substrates (Kuzyakov, 2002). The release of low-molecular-weight C compounds by plant roots increases the capability of otherwise energy-limited saprotrophic soil microbes to produce extracellular enzymes for degradation of high-molecular-weight organic compounds. This in turn results in increased availability of labile organic and inorganic nitrogen (N) and phosphorus (P) to both soil microbes and plants.

When plants are in mycorrhizal symbioses, however, a substantial proportion of plant photosynthates is allocated to mycorrhizal fungi (Hobbie, 2006; Smith and Read, 2008), which deliver nutrients in return. Mycorrhizal hyphae produce extensive extraradical mycelia (Agerer, 2001), which increase the plant's accessible soil volume substantially, making resources from distant soil regions available for plants. The two most prevalent mycorrhizal types, arbuscular mycorrhiza (AM) and ectomycorrhiza (ECM), differ fundamentally regarding their nutrient acquisition strategies (Chapman et al., 2005; Phillips et al., 2013). AM fungi have no or only limited capabilities to produce extracellular enzymes for degrading complex organic matter (Smith and Read, 2008), yet they have been shown to promote decomposition of organic material and utilize thereby released N (Hodge et al., 2001). Consequently, it has been suggested that AM fungi rely on associated saprotrophic microorganisms to gain access to nutrients bound in organic matter (Hodge et al., 2010; Jansa et al., 2013). Transfer of plant-derived C to bacteria via AM hyphae has been shown in a range of studies (Toljander et al., 2007; Drigo et al., 2010; Cheng et al., 2012; Kaiser et al., 2015; Paterson et al., 2016), indicating that *hyphosphere priming* with plant photosynthates by AM hyphae may be a relevant process for C cycling in AM-dominated ecosystems (Talbot et al., 2008).

In contrast to AM fungi, ECM fungi are known to produce a range of extracellular enzymes to degrade complex organic compounds (Read et al., 2004; Talbot et al., 2008). However, to what extent they possess saprotrophic capabilities (Bruns et al., 1998), and if the involved genes are expressed when the fungus is in a mycorrhizal symbiosis (Pellitier and Zak, 2017) is not fully understood. Specific ECM fungal lineages, depending on their enzymatic repertoire, may or may not depend on free-living saprotrophs for SOM nutrient liberation. Thus, while AM fungi are likely to cooperate with free-living soil saprotrophs by supplying them with plant-derived C, and receiving nutrients in return, the hyphospheric interactions of ECM fungi are much less clear.

Interactions between symbiotic partners have been suggested to be reciprocal as this ensures evolutionary stability (Noë and Hammerstein, 1995; Kiers and Van der Heijden, 2006). Hence, if mycorrhizal fungi rely on free-living saprotrophs for nutrient acquisition, their resource exchange would likely be reciprocal, where the amount of resource provided depends on the amount of resources received. In this sense, mycorrhizal fungal hyphae, which are able to explore the soil's pore space, may supply increased amounts of labile C to soil saprotrophs at spots with higher availability of nutrient-rich breakdown products or inorganic nutrients in order to further stimulate decomposition of a potential organic matter point source. Given that organic matter most likely exhibits a patchy distribution at the soil's microscale, this may be a viable strategy for nutrient exploration by adjusting the extent of C exudation to the amount of organic matter available at certain spots in the soil.

As a first step toward a better understanding of the interaction of ECM fungi and free-living soil saprotrophs and to elucidate whether hyphosphere priming is an integral process in ECM systems, it needs to be clarified (i) whether there is a transfer of recently photoassimilated plant C to soil bacteria via ECM hyphae, and (ii) whether this transfer is controlled by soil nutrient conditions.

To address these questions we investigated the short-term flow of plant-assimilated C and fungal-obtained soil N through an ECM system. For this purpose, we conducted a dual stable isotope labeling experiment with young beech (*Fagus sylvatica* L.) trees. Plants were grown in “split-root” pots consisting of two soil compartments, dividing the root system into two parts (Bever et al., 2009). Each of the soil compartments was connected to a root-inaccessible litter compartment, which was accessible by mycorrhizal hyphae only. Plant canopies were exposed to a ^13^C-CO_2_ labeled atmosphere for several hours (“pulse-chase experiment”). This setup allowed us to trace the flow of recently photoassimilated C from plants via ECM hyphae to soil bacteria using phospholipid fatty acid (PLFA) stable isotope probing as well as nano-scale secondary ion mass spectrometry (NanoSIMS) isotope imaging. Furthermore, we added a mixture of ^15^N labeled ammonium and amino acids to one of the two litter compartments to investigate the influence of N availability on the relationship between ECM hyphae and soil bacteria. We hypothesized that (i) ECM fungi transfer plant-derived C to associated hyphosphere bacteria and that (ii) a higher local availability of labile N compounds in soil increases hyphal exudation of plant C.

## Materials and Methods

### Experimental Setup: Split-Root System With Hyphae-Exclusive Compartment

We collected 3- to 4-year-old beech (*Fagus sylvatica* L.) trees in a temperate beech forest site *ca*. 40 km south-west of Vienna, Austria (510 m above sea level) in July 2014. Additionally, we sampled soil (uppermost 5 cm, A horizon) and beech litter. The soil at this site is a dystric cambisol (over flysh) with a pH of 4.5–5.1 (Kaiser et al., 2010, 2011). Soil was sieved for homogenization (4 mm) and mixed with perlite (soil:perlite = 8:1, v/v) to enhance aeration, a method that has been shown to improve growing conditions for ECM fungi (Peter Schweiger, unpublished data). The litter was carefully homogenized by hand. Mycorrhization of the collected plants was confirmed by visual inspection under stereo microscopes.

**Figure 1:**
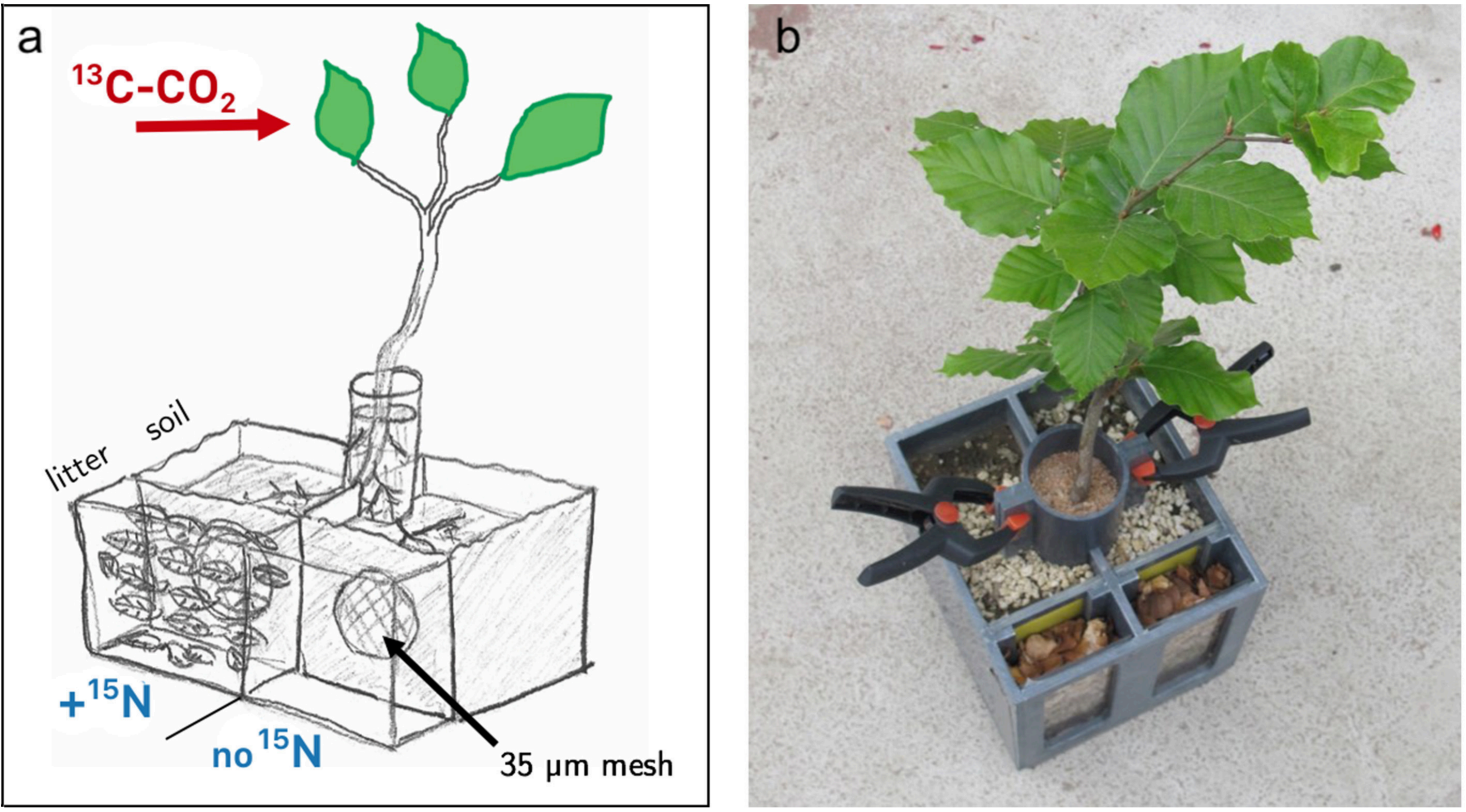
Design of split–root boxes. Plants grew in two separated soil compartments where the root system was divided into two halves. Stems were stabilized in a cylinder filled with quartz sand. Each soil compartment was connected to a leaf litter compartment, separated by a mesh (35 μm) which is penetrable by fungal hyphae, but not by roots. Plant canopies were exclusively exposed to a ^13^C-CO_2_ enriched atmosphere. A mixture of ^15^N-labeled ammonium and amino acids was added to only one litter compartment per box. **(a)** Schematic drawing of the setup. One of the two litter compartments is drawn without litter to illustrate the chamber design with the separating mesh. For the experiment both outer compartments were filled with beech litter. **(b)** Photo of a plant used in the labeling experiment.

Plants were potted in “split-root”-boxes (each 114 × 60 × 125 cm), which allowed to divide and subsequently manipulate the root system in separated soil compartments. In order to ensure an even distribution of roots in the two compartments, each plant was first freed of soil, and its root system was carefully investigated. If the plant had a dominant primary root, it was cut allowing the root system to be split evenly above the cut surface. If its primary root was bifurcating, the roots were pruned below the branch, thus creating root systems of comparable size. To compensate for the considerable loss of roots, the trees were trimmed below the first aboveground stem branch, resulting in leafless trees. We then transplanted the trees into the split-root boxes, carefully dividing the root system into the two compartments, which were filled with 750 ml of the aforementioned soil-perlite mixture, and the stems were stabilized by attaching a cylinder filled with sterile quartz sand (Figure 1). Each soil compartment was connected to another compartment (10 × 60 × 125 cm) filled with homogenized leaf litter (26 g compartment^−1^). These “litter” compartments were separated from the respective soil compartments by a mesh (35 μm) which could be penetrated by fungal hyphae but acted as a barrier to plant roots (Figure 1). There was no connection between the two litter compartments. Plants were allowed to grow in the split-boxes for 12 months in a glasshouse under natural sunlight, temperature, and otherwise controlled conditions. The glasshouse was equipped with a movable ceiling, which remained open unless rain or strong winds were registered. Soil and litter compartments were watered regularly and equally on both sides of the boxes.

### Dual Isotope Labeling Experiment

To trace plant N uptake via the mycorrhizal pathway, 12 ml of a ^15^N-labeled solution of ammonium (98 atom% ^15^N-NH_4_Cl, Sigma Aldrich, Vienna, Austria; 54.5 mg/l, i.e., 1 mM N) and amino acids (Algal Amino Acid mixture, U-^15^N 98 atom%, Cambridge Isotope Laboratories, Cambridge, UK; 140 mg/l, *ca*. 1 mM N based on an assumed average amino acid molar weight of 140 g/mol), dissolved in water, were added to one of the two litter compartments on average 48 h prior to sampling. This time period was selected based on preliminary tests. To ensure equivalent conditions, 12 ml of water was added to the other litter compartments. Diffusion between the soil and litter compartments was prevented by the structure of the mesh, which consisted of two membranes with an intermediate air-filled space. Elevating the “split-root” boxes on wooden blocks prevented water transfer between plant boxes.

Twenty-four hours after ^15^N addition, aboveground plant parts were incubated in a ^13^C-CO_2_ enriched atmosphere (~90 atom% ^13^C; 1,500 ppm) for 6 h 20 min using a gas-tight acrylic glass incubation chamber (1,350 × 545 × 320 cm; Figure S1). The chamber is mounted on four vertically positioned acrylic glass plates (545 × 190 cm) so that pots can be placed beneath it. The top cover of the cuboid chamber can be lifted from the bottom plate. Lengthwise, the bottom plate consists of three separate parts, so that the outer parts can be removed by sliding them outwards. Opposite half-circle cutouts at the interface between middle and outer plates allow to fit in plant stems before closing the plates. Remaining gaps around the stems were sealed with Terostat^®^ adhesive before the chamber was closed by mounting the top cover. Thus, plant canopies were exclusively exposed to chamber atmosphere, while soil and litter compartments were isolated from the incubation chamber. Additionally, any potential photosynthesis in the split-root boxes was inhibited by wrapping the boxes with black plastic bags (Figure S1).

^13^C-CO_2_-labeling took place under natural daylight conditions (cloudy, partly sunny) on 16^th^ June 2015. Plants were arranged as described above, and the chamber was closed at noon. Inside chamber temperature stayed between 21 and 24°C in the course of the labeling procedure. Two small fans installed inside the chamber ensured a homogeneous atmosphere. Initial CO_2_ concentrations were monitored using an infrared gas analyzer (EGM-4, PP systems, Hitchin, UK) connected to the chamber, showing that CO_2_ was decreasing rapidly inside the chamber due to photosynthetic activity. Sixty milliliters of ^13^C-CO_2_ (99 atom%, Sigma-Aldrich, Vienna, Austria), corresponding to 255 ppm in the chamber volume, were injected with a gas-tight syringe via built-in septa every 30 min. This approach repeatedly replenished CO_2_ withdrawn by plant photosynthesis, leading to a quick rise of ^13^C enrichment of CO_2_ in the chamber. Gas samples were drawn from the chamber at regular intervals starting 1.5 h after chamber closure, and later analyzed by a headspace gas sampler (GasBench II, Thermo Fisher Scientific, Bremen, Germany) interfaced to continuous-flow isotope ratio mass spectrometry (Delta Advantage V, Thermo Electron, Bremen, Germany) to determine total CO_2_ concentration and ^13^C enrichment in the chamber during the course of the experiment (85.5 ± 1.8 atom% ^13^C (average ± SE), 875 to 3,300 ppm CO_2_).

After the ^13^C-CO_2_-labeling, plants were kept overnight in the dark and harvested in a random sequence within a timeframe of 10 h over the following day (i.e., 19–29 h after the start of the 6 h labeling period, referred to as *ca*. 24 h after labeling from now on). Here, we distinguished between leaves, stem, roots, rhizosphere soil, bulk soil (soil compartment), and litter (litter compartment). Bulk soil was defined as the soil remaining in the split-root boxes upon the removal of the plants, including soil which fell off the roots when shaking the plant gently. Consequently, rhizosphere soil was defined as the soil that remained attached to the roots after shaking, and was carefully removed by hand. Meshes were checked for intactness and root breakthrough.

Eight plants received ^15^N- and ^13^C-CO_2_-treatments, four plants only the ^13^C-CO_2_-treatment, four plants were unlabeled controls, and four unlabeled boxes were kept without plants. One of the double-labeled plants had to be excluded from all analyses, because root-biomass on one side was extremely low (*n* = 7, Table S1). PLFA analysis was carried out on a subset of 6 replicates of the valid double-labeled plants (*n* = 6). Unlabeled controls plants served as a natural abundance control, and were used to calculate ^13^C excess values. Plants that were only ^13^C-labeled and unplanted boxes were used to rule out a systematic error in differences between the two litter compartments caused by other factors than N addition. Data from these samples are not explicitly shown. Unplanted boxes were also used as a control for assessing the mycorrhizal fungal community in litter compartments that originated from mycorrhizal fungi associated with plants in the planted boxes (see section *Mycorrhization and Identification of Fungal Communities via Sequencing of Internal Transcribed Spacer 1 (ITS1) Region*).

In order to assess the flow of photoassimilated ^13^C through soil and hyphae-exclusive litter, we measured ^13^C of dissolved organic carbon (DOC), microbial biomass, and PLFAs in the respective compartments. Unlabeled control plants served as natural abundance control. Comparison of the two split-root box sides of plants only labeled with ^13^C-CO_2_ ensured that equivalent conditions were present.

### Mycorrhization and Identification of Fungal Communities Via Sequencing of Internal Transcribed Spacer 1 (ITS1) Region

Immediately after plant harvest, roots from each soil compartment were split into up to five parts (depending on the size of the root) which were analyzed under stereo microscopes for their degree of mycorrhization. Due to time-constraints during harvest (all plants needed to be processed within a few hours to ensure similar residence times of labeled C and N) mycorrhization (i.e., percentage of root tips colonized by ECM fungi) was roughly estimated based on visual screening of each of the root parts with the stereo microscope. The aim of this approach was not to get an accurate measure of mycorrhizal colonization, but a confirmation that roots of each plant were largely colonized (on average, estimations were at 85%). Roots were additionally scanned to allow documentation of their mycorrhizal status (for one example, see Figure S2).

Rhizosphere samples for downstream molecular analysis were sampled by cutting root pieces from the root after shaking off the bulk soil. Root pieces were transferred into 50 ml tubes containing 1x phosphate-buffered saline (pH = 8). After 30 min of shaking, the root was transferred into a fresh tube and the remaining slurry was centrifuged (4 min at 8,000 x g). The supernatant was discarded and the pellet (considered rhizosphere soil) was stored at −80°C until extraction. For sequencing of ITS1 regions, DNA was extracted from 400 mg of litter and rhizosphere soil samples using a phenol-chloroform protocol (Angel, 2012) and quantified using the Quant-iT™ PicoGreen® dsDNA Assay Kit (Thermo Fisher, Waltham, USA). The ITS1 region was amplified from these DNA samples via polymerase chain reaction (PCR) using multiplexed barcoded amplicon sequencing on the Illumina MiSeq platform (Illumina, San Diego, USA), as described by Herbold et al. (2015). The fungal ITS1 region was amplified in triplicate reactions using 30 cycles with primers ITS1F (5′ CTT GGT CAT TTA GAG GAA GTA A 3′, Gardes and Bruns, 1993) and ITS2 (5′ GCT GCG TTC TTC ATC GAT GC 3′, White et al., 1990, used as the reverse primer). Each 20 μl PCR reaction contained 13.95 μl nuclease free water, 2 μl 10x DreamTaq Green Buffer, 2 μl 2 mM dNTP mix, 0.125 μl BSA (0.1 μg μl^−1^), 0.125 μl of 1.25U DreamTaq Green DNA Polymerase, 0.4 μl 0.2 μM of each primer, and 1 μl DNA template (*ca*. 10 ng per reaction). The following thermocycling conditions were utilized for the first step PCR: 94°C for 3 min, 94°C for 45 s, 52°C for 60 s, 72°C for 90 s, and a final step of 72°C for 10 min. The triplicate PCR products were pooled, assessed for the correct product size by gel electrophoresis visualized with GelRed Nucleic Acid Stain (Biotium Inc., Fremont, USA) and purified using the ZR-96 DNA Clean-up kitTM (Zymo Research, Irvine, USA). A second PCR reaction (additional 8 cycles) with the purified first step PCR product along with primers containing sample-specific barcodes was used for subsequent pooling prior to sequencing (Herbold et al., 2015). The second step PCR was carried out in 50 μl reactions containing 32.5 μl nuclease free water, 5 μl 10x DreamTaq Green Buffer, 5 μl 2 mM dNTP mix, 0.25 μl BSA (0.1 μg μl^−1^), 0.25 μl of 1.25U DreamTaq Green DNA Polymerase, 4 μl of barcode (0.8 μM), and 3 μl of the purified first step PCR reaction. The correct product size was verified by gel electrophoresis as described above and purified using the ZR-96 Clean-Up Kit (Zymo Research). Final products were quantified by Quant-iT™ PicoGreen® dsDNA Assay Kit and pooled equimolarly to 20 x 10^9^ copies per sample library.

Sequencing was performed at Microsynth AG (Balgach, Switzerland). The TruSeq Nano DNA Library Prep Kit (Illumina, San Diego, USA; Protocol Illumina, FC-121-4003-excluding the fragmentation step) was used for the adaptor ligation. The MiSeq Reagent kit v3 (Illumina) was used with a run configuration of 2 x 300 cycle.

Paired MiSeq reads were split into sample datasets as described previously (Herbold et al., 2015). The paired-end raw fungal ITS1 MiSeq reads were extracted from raw amplicon data using ITSx (Bengtsson-Palme et al., 2013) and singletons were removed. Fungal OTUs were identified according to 99% sequence similarity threshold and the taxonomic classification was implemented by a Naïve Bayesian sequence classifier (Wang et al., 2007) using the Warcup training set version2 (Deshpande et al., 2016) at a confidence cutoff of 80%. Reads related to ECM fungi were identified based on literature (https://mycorrhizas.info/ecmf.html; Tedersoo and Smith, 2013). The sequence data were deposited in the NCBI Short Read Archive under study accession number PRJNA518765.

### Tracing Plant-Derived Carbon and Hyphal-Assimilated Nitrogen in Bulk Pools

^13^C/^12^C and ^15^N/^14^N isotopic ratios in bulk pools were measured with an elemental analyzer (EA 1110 elemental analyzer, CE Instruments, Milan, Italy) coupled to an isotope-ratio mass spectrometer (Finnigan MAT DeltaPlus, Thermo Finnigan, Bremen Germany).

### ^13^C, Carbon and Nitrogen in Microbial Biomass and Dissolved Pools

To determine DOC, total dissolved N (TDN) and C and N in microbial biomass, 1.5 g of fresh soil and litter samples were extracted with 15 ml of 0.5 M K_2_SO_4_ before and after chloroform fumigation extraction (CFE; Brookes et al., 1985; Vance et al., 1987). Aliquots of the extracts were measured on a TOC/TN analyzer (TOC-V CPH/TMN-1, Shimadzu, Japan). Microbial biomass was calculated as the difference between fumigated and unfumigated samples. We did not include biomass conversion factors into our calculations. ^13^C in DOC of fumigated and unfumigated samples was measured on a high performance liquid chromatography system (ICS-3000 Single Pump SP-1, Dionex, Sunnyvale, CA, USA) interfaced with an isotope ratio mass spectrometer (Finnigan Delta-V, Thermo Electron, Germany) through an LC-Isolink (Thermo Electron, Germany). We injected directly (i.e., without column) at a flow of 0.5 ml ultrapure water min^−1^ (Millipore, Vienna, Austria). ^13^C in microbial biomass was calculated as the difference in atom% ^13^C between fumigated and unfumigated samples. ^13^C–enrichment was calculated as the difference in relative isotopic enrichment between labeled and unlabeled control samples as atom% excess ^13^C (Kaiser et al., 2015).

### Phospholipid Fatty Acids

Total lipids were extracted from soil and litter samples with a mixture of chloroform, methanol and citrate buffer (1:2:0.8, v/v/v; Bligh and Dyer, 1959). After fractionation on silica columns, collected phospholipids were derivatized to methyl esters via alkaline methanolysis (Frostegård et al., 1991) and dried under a constant stream of N_2_. Dry fatty acid methyl esters (FAMEs) were re-dissolved in iso-octane and subsequently measured on a gas chromatograph (Trace GC Ultra, Thermo Scientific, Germany) coupled to a mass spectrometer (ISQ, Thermo Scientific, Germany) for PLFA identification, and on a Trace GC-Ultra (Thermo Fisher Scientific, Milan, Italy) coupled to a Delta V Advantage isotope ratio MS (Finnigan Delta-V, Thermo Fisher Scientific, Bremen, Germany) via a GC IsoLink (Thermo Fisher Scientific, Bremen, Germany) for quantification of the relative abundance and determination of isotopic ^13^C/^12^C ratios of PLFAs. Non-adecanoic acid (FAME 19:0) was added to samples prior to methylation and used as an internal standard for quantification. Bacterial and fungal fatty acid methyl esters (BAME CP mix, Supelco; 37 Component FAME mix, Supelco) were used as qualitative standards. ^13^C enrichment was corrected for the isotope ratio of methanol-C added with methanolysis.

PLFAs were assigned to phylogenetic groups as reported by Ruess and Chamberlain (2010) unless noted otherwise. The PLFAs i15:0, a15:0, i16:0, i17:0, a17:0 were used as indicators for Gram-positive bacteria in general, 10Me17:0 (Zelles, 1999) for Actinobacteria specifically, 16:1ω5, 16:1ω7, cy17:0, and cy19:0 for Gram-negative bacteria, 17:0 (Hill et al., 2000; Koranda et al., 2011; Schnecker et al., 2012) and the sum of the above for bacteria in general, cis18:1ω9, trans18:1ω9 and 18:2ω6,9 for fungi, and 16:0 and 18:0 for general PLFAs (Zelles, 1999; Leckie, 2005). The PLFA 16:1ω5 is also known to occur in AM fungi (Olsson, 1999). However, since *F. sylvatica* is not known to associate with AM fungi, 16:1ω5 represents most likely a biomarker for Gram-negative bacteria in this experiment (Phillips et al., 2002). The fungal PLFAs 18:1ω9 and 18:2ω6,9 were analyzed separately since they can vary considerably between fungal species (Zelles, 1997; Olsson, 1999).

We calculated isotopic enrichment in individual PLFAs as the difference in relative ^13^C-enrichment of labeled samples to unlabeled controls as atom% excess ^13^C (Kaiser et al., 2015). To calculate the relative ^13^C content in respective microbial groups, relative ^13^C-enrichment was weighted by the mean abundance (in μg C in PLFAs g^−1^ dry soil) of individual PLFAs.

### Scanning Electron Microscopy and NanoSIMS

Scanning electron microscopy (SEM) was used to evaluate preparation strategies for optimum structural preservation of hyphae collected from the litter compartments, and to screen individual hyphal samples for their suitability for NanoSIMS measurements. SEM images, representing the signal intensity distribution of secondary electrons, were acquired with a SEM Jeol IT 300 (JEOL, Freising, Germany). Several combinations of different fixation and drying techniques were tested to find the optimum preparation procedure aimed at preserving the structure of the hyphae and sustaining attachment of associated microorganisms. In these tests, unfixed or chemically fixed (using formaldehyde or glutaraldehyde-osmiumtetroxide) hyphae were either air dried, dehydrated for critical point drying or treated with hexamethyldisilazane as a drying agent. These tests showed that air-drying yielded best results in preserving the structure of hyphae and the association of attached microorganisms. As air drying is also advantageous in terms of minimizing loss of diffusible compounds (transport forms of recent photosynthates and N in ECM hyphae are most likely sugars and amino acids; Martin and Botton, 1993; Taylor et al., 2004) compared to chemical fixation, we chose this preparation method for combined SEM and NanoSIMS analysis of fungal hyphae.

Hyphae were sampled and prepared for NanoSIMS analysis as follows: Litter compartments (from boxes exposed to stable-isotope labeled substrates as well as unlabeled control boxes) were screened for hyphae utilizing stereo microscopes. Per compartment, three to five hyphal bundles attached to leaves were collected with tweezers. Hyphal bundles were carefully and quickly dipped in H_2_O_MQ_ to remove soil particles. One to two single hyphae were placed in fresh H_2_O_MQ_ droplets spotted on Vectabond^TM^ (Vector Laboratories Inc., Burlingame, USA) reagent-coated, antimony-doped silicon wafer platelets (7.1 × 7.1 × 0.75 mm, Active Business Company, Brunnthal, Germany) and air-dried. Samples were sputter-coated with gold at 80 mA for 100 s using a High Resolution Fine Coater JFC-2300 HR (JEOL, Freising, Germany), yielding thin gold films of ~20 to 40 nm thickness.

Fungal hyphae were critically screened by SEM to identify regions appropriate for NanoSIMS analysis. To measure the isotope content within the fungal hyphae (i.e., in the cell lumen), we screened for single, plane hyphae exhibiting a clean surface devoid of soil particles and microbial cells, located preferentially in the center of the silicon wafer platelets. For the NanoSIMS analysis of hypha-colonizing microorganisms, samples were screened by SEM for plane hyphal surfaces containing attached microorganisms. From 20 screened fungal hyphae, one was chosen representative for each sample type. Due to the labor-intensive nature of NanoSIMS analysis on these specific samples, we only had the chance to acquire data from one hypha per sample type. Consequently, we do not claim that these are representative measurements, but instead consider them as supporting data embracing spatial structure and thus providing topochemical information.

NanoSIMS measurements were performed on a NanoSIMS 50L (Cameca, Gennevilliers, France) at the Large-Instrument Facility for Advanced Isotope Research at the University of Vienna. In order to minimize degradation of the mass resolving power (MRP) due to topography, samples were mounted with a preferentially horizontal alignment of fungal hyphae inside the NanoSIMS analysis chamber. Prior to data acquisition, analysis areas were pre-sputtered utilizing a high-intensity, slightly defocused Cs^+^ ion beam (100 pA beam current, ~1 μm spot size). Data were acquired as multilayer image stacks by sequential scanning of a finely focused Cs^+^ primary ion beam (*ca*. 80 nm probe size at 2 pA beam current) over areas between 40 × 40 and 52 × 52 μm^2^ at 512 × 512 pixel image resolution and a primary ion beam dwell time of 15 to 20 msec/(pixel^*^cycle). For enhancement of the measurement efficiency, imaging of individual hyphae with horizontal alignment was performed under vertical confinement of the scanning area. ^12^C^−^, ^13^C^−^, ^12^C^12^C^−^, ^12^C^13^C^−^, ^12^C^14^N^−^, ^12^C^15^N^−^, ^31^P^−^ secondary ions as well as secondary electrons were detected simultaneously.

Images based on NanoSIMS measurement data were generated using the Open MIMS plugin (Poczatek et al., 2009) in the FIJI package based on ImageJ (Schindelin et al., 2012). C isotope composition images displaying the ^13^C/(^12^C+^13^C) isotope fraction, designated as atom% ^13^C, were inferred from the C^−^ and ${\text{C}}_{2}^{-}$ secondary ion signal intensity distribution images via per-pixel calculation of ^13^C^−^/(^12^C^−^+^13^C^−^) and ^13^C^12^C^−^/(2·^12^C^12^C^−^+^13^C^12^C^−^) intensity ratios. N isotope composition images displaying the ^15^N/(^14^N+^15^N) isotope fraction, designated as atom% ^15^N, were inferred from the ^12^CN^−^ secondary ion signal intensity maps via per-pixel calculation of ^12^C^15^N^−^/(^12^C^15^N^−^+^12^C^14^N^−^) intensity ratios. For visualization of relative phosphorus and nitrogen elemental distributions, C (i.e., matrix) associated secondary ion signals were utilized as reference signals. As such, relative phosphorus-to-carbon ratios, designated as (P/C)_rel._ given in arbitrary units [a.u.], were inferred from C^−^ normalized ^31^P^−^ signal intensities via ^31^P^−^/(^12^C^−^+^13^C^−^). Correspondingly, relative nitrogen-to-carbon elemental ratios, designated as (N/C)_rel._ given in arbitrary units [a.u.], were obtained from ${\text{C}}_{2}^{-}$ normalized CN^−^ signal intensities. Overlay images, combining morphological with chemical information, were assembled using GIMP 2.10.4 (GNU Image Manipulation Program, https://www.gimp.org/). SEM (secondary electron) images where utilized for representation of the sample morphology prior to NanoSIMS analysis. For visualization of the sample morphology evolving during NanoSIMS imaging, C^−^ signal intensity distribution images were used.

Region of interest (ROI) specific numerical data evaluation was conducted using the WinImage software package (version 2.0.8) provided by Cameca. ROIs, referring to individual microbial cells (**Figure 7**), were manually defined based on the relative N/C and P/C elemental ratio maps (serving as indicators for biomass) and cross-checked by the morphological appearance in the C^−^ secondary ion intensity distribution and SEM images (Figure S3). Within the fungal lumen (**Figure 5**), ROIs were defined according to the isotope enrichment patterns since it was not possible to identify characteristic cellular features. This was likely because luminal regions were accessed via depth-profiling, which is less suited for visualization of cellular (ultra)structure than e.g., cross-sectional analysis of resin embedded samples. C and N isotope compositions were calculated from the accumulated intensities of ^12^C^12^C^−^ and ^12^C^13^C^−^ and ^12^C^14^N^−^ and ^12^C^15^N^−^ secondary ion signals detected within each ROI. The isotopic composition values summarized in the boxplot shown in **Figure 6** were determined by averaging over the individual images of the multilayer stack (ranging from 16 to 24 individual cycles). A more detailed description of the NanoSIMS measurement approach, including the concepts for data evaluation and visualization, is provided in the Supplementary Methods.

### Statistical Analysis

R (version 3.4.3) was used for all statistical analyses (R Core Team, 2017), with the packages “ggplot2” for plotting (Wickham, 2009) and “vegan” for multivariate analysis (Oksanen et al., 2018). Means or medians were considered to be significantly different from each other when *p* < 0.05. Since data transformations did not result in normal distribution or homogeneity of variance in all samples, non-parametric tests were used in all statistical analyses. Labeled samples were compared to unlabeled controls with the Mann-Whitney *U*-test to validate significant ^13^C-enrichment. Since N-treated and untreated plant box sides were under the influence of roots from the same plant for >1 year, they violated the independence assumption of many statistical tests; thus, plant box sides were compared with the Mann-Whitney *U*-test for paired samples. Pools were compared with the Kruskal-Wallis rank sum test optionally followed by Dunn's *post-hoc* test of multiple comparisons with Bonferroni correction.

ROI data extracted from NanoSIMS isotope composition images were tested for normal distribution using the Kolmogorov-Smirnov test and significance of enrichment in comparison to the natural abundance control was tested using the Welch *t*-test.

## Results

### Evidence of Ectomycorrhizal Fungi Occurrence in Litter and Rhizosphere Compartments

**Figure 2:**
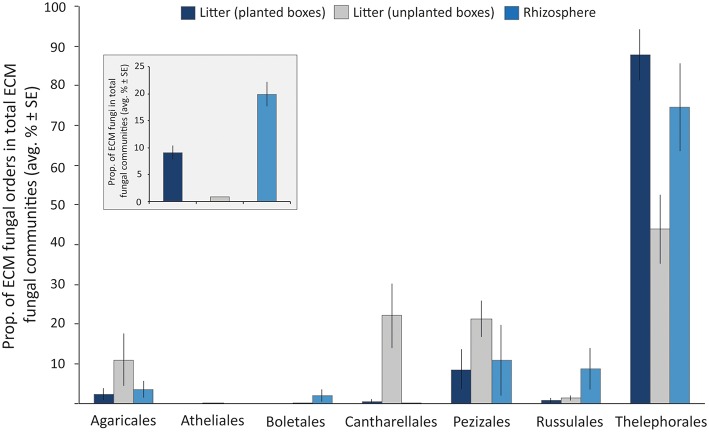
Bar graph illustrating the proportion of ECM fungal orders in total ECM fungal communities in litter samples derived from planted (dark blue) and unplanted boxes (gray), as well as from the rhizosphere soil (light blue) as average proportion of the libraries ± standard error (SE). The insert graph depicts the proportion of ECM fungi in the total fungal communities (average proportion of the libraries ± SE).

ITS1 libraries of the litter (derived from 7 planted boxes and 4 unplanted boxes) and rhizosphere (*n* = 7) samples contained a total of 148,156 reads separated into 4,721 OTUs. These OTUs were comprised of Ascomycota and Basidiomycota spanning a variety of different orders, such as Dothideomycetes, Sordariomycetes, Leotiomycetes (Ascomycota) and Agaricomycetes, Tremellomycetes (Basidiomycota) among others. The Zygomycota were mainly represented by members of the order Mortierellales. Among the total fungal reads, members of the ECM fungi were detected in both, the rhizosphere and litter samples of planted boxes, at considerable proportions (19.87 ± 2.26% (average ± SE) and 9.04 ± 1.26%, respectively (Figure 2). Furthermore, 73% of the OTUs detected in the litter ECM fungal communities (excluding singletons) were detected in the rhizosphere ECM fungal communities. In contrast, the ECM fungi only comprised 0.8 ± 0.04% of the fungal communities in the litter samples of unplanted boxes. The average proportion of the orders in the ECM fungal communities are depicted in Figure 2. Members of the order Thelephorales were the dominating ECM fungi in our system, followed by Pezizales, Cantharellales, Agaricales, Russulales, Boletales, and Atheliales. Thelephorales constituted on average 87% of the ECM fungal communities in litter samples derived from planted boxes, compared to 44% in unplanted boxes and 75% in the rhizosphere. The proportion of Agaricales, Cantharellales and Pezizales in the ECM fungal community was highest in litter samples of unplanted boxes (up to 22%) compared to planted boxes, while the proportion of the Russulales and Boletales was highest in rhizosphere samples.

### Bidirectional Transfer of Recently Photoassimilated C and N Taken Up by Ectomycorrhizal Hyphae

**Figure 3:**
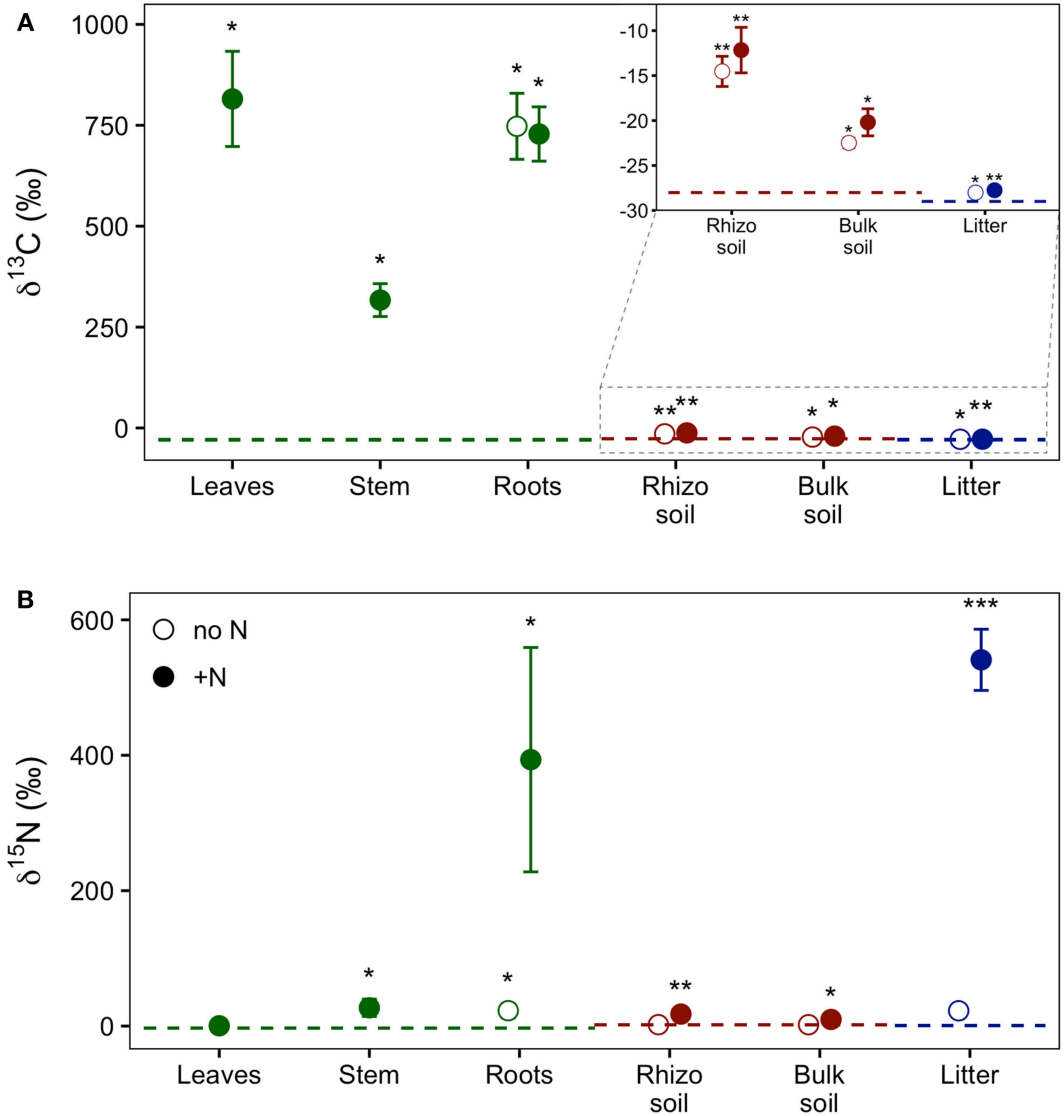
Relative enrichment of **(A)** ^13^C and **(B)** ^15^N isotopes in plant, soil, and litter samples 24 h after ^13^C-CO_2_ labeling of plants and 48 h after addition of ^15^N-labeled NH_4_ and amino acids to litter compartments, indicating transfer of these supplies through the experimental system. Colors indicate the experimental system parts, i.e., plant (green), soil (red), and litter (blue). Dashed lines represent natural abundance values (mean of the respective system part). Symbols show results from dual-labeled (^13^C and ^15^N) plant boxes. Open circles, untreated side of the box; closed circles, N-treated side. This “side” designation refers only to roots, litter, and soil, as aboveground plant parts (i.e., leaves and stem) are connected to both sides. Error bars represent standard error. Asterisks indicate significant difference from natural abundance (^*^p < 0.05, ^**^p < 0.01, ^***^p < 0.001; Mann-Whitney *U*-test; *n* = 7).

**Figure 4:**
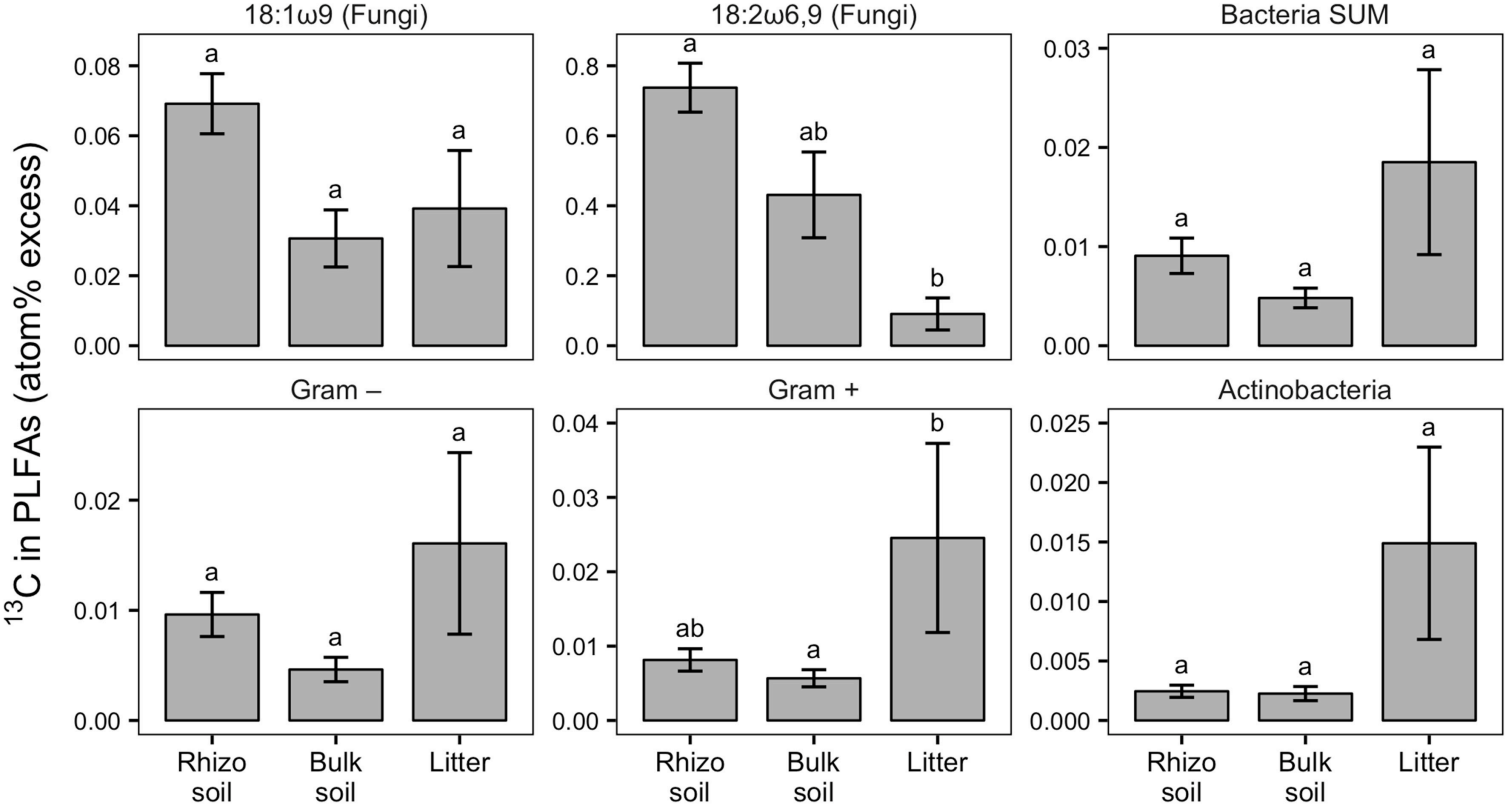
Relative enrichment of ^13^C in fungal-specific (18:1ω9 and 18:2ω6,9) and bacteria-specific PLFAs in rhizosphere, bulk soil, and litter pools shown as atom% excess ^13^C (calculated by subtracting the natural abundance atom% value of the respective PLFA biomarkers). Bars show the weighted mean of each pool from N-treated and untreated sides from dual-labeled (^13^C and ^15^N) plant boxes. This format was chosen to emphasize general trends of relative ^13^C enrichment in fungal and bacterial groups across the different pools irrespective of N addition. No significant differences in atom% excess ^13^C could be detected between the N-treated and the untreated sides (+N/noN; Mann-Whitney *U*-test for paired samples). For visualization of differences between +N/noN box sides see Figure S5. Litter compartment enrichment indicates allocation of ^13^C via ECM hyphae to root-distant bacteria. Error bars represent the standard error; *n* = 6. Differences in pools were analyzed with the Kruskal-Wallis rank sum test. Significant tests (*p* < 0.05) were followed by Dunn's *post-hoc* test of multiple comparisons with Bonferroni correction (adj. *p* < 0.05); significant differences are indicated by lowercase letters. All microbial groups were significantly enriched in ^13^C in all pools, except for 18:2ω6,9, Gram-negative bacteria and Actinobacteria, each in bulk soil (comparison of atom% ^13^C values via Mann-Whitney *U*-test, *p* < 0.05; not shown).

Our experimental setup allowed the simultaneous tracing of photoassimilated C and N available for fungal hyphae in an ECM symbiosis, demonstrating a rapid exchange of these resources between the symbiotic partners. Twenty-four hours after the start of the 6-h ^13^C-CO_2_ exposure a significant fraction (11.74%) of the ^13^C photoassimilated by the plants had been transferred belowground into soil and litter pools (Table 1, Figure 3A). Moreover, a substantial fraction (3.0%) of the total ^13^C allocated outside the plant roots was delivered to the root-inaccessible litter compartment. In addition, microbial biomass, based both on CFE (Table 2) and fungal and bacterial PLFA biomarkers, was significantly ^13^C enriched in this compartment (Figure 4, Table S2), indicating a rapid hyphal transport. Interestingly, the two fungal-specific PLFAs 18:1ω9 and 18:2ω6,9 differed in their incorporation of ^13^C between rhizosphere, bulk soil and litter pools: while isotopic enrichment of ^13^C was similar for 18:1ω9, it decreased significantly from rhizosphere soil to litter for 18:2ω6,9.

**Table 1:** Total C, ^13^C, N, and ^15^N per box (in absolute values), percent of total ^13^C and ^15^N showing allocation of recent photosynthates and N from the litter compartment in bulk pools, and C/N ratio of bulk pools.

**Pool**	**C (g)**	**^**13**^C excess (mg)**	**% ^**13**^C** **of total ^**13**^C**	**N (g)**	**^**15**^N** **excess (μg)**	**% ^**15**^N of total ^**15**^N**	**C/N**
Leaves	0.49 (0.05)	4.4 (0.64)	20.86 (2.74)	0.03 (0.00)	0.32 (0.14)	0.10 (0.05)	19.01 (0.49)
Stem	2.07 (0.19)	7.41 (0.48)	35.92 (2.21)	0.03 (0.00)	3.72 (1.73)	1.21 (0.58)	62.74 (4.09)
Roots	0.88 (0.14)	6.57 (0.74)	31.47 (3.04)	0.02 (0.00)	16.11 (4.66)	5.71 (1.99)	36.98 (1.37)
**Plant biomass**	3.44 (0.31)	18.37 (1.10)	88.26 (1.35)	0.08 (0.01)	20.15 (5.09)	7.02 (2.12)	-
Rhizosphere soil	6.70 (1.29)	0.96 (0.22)	4.35 (0.76)	0.53 (0.10)	13.22 (3.41)	4.25 (1.12)	12.46 (0.14)
Bulk soil	25.64 (1.88)	1.52 (0.34)	7.04 (1.25)	2.05 (0.16)	35.77 (13.48)	10.25 (3.07)	12.51 (0.06)
Litter	6.23 (0.15)	0.08 (0.03)	0.36 (0.13)	0.24 (0.00)	250.48 (18.46)	78.48 (3.56)	25.7 (0.39)
**Soil** **+** **Litter**	38.57 (1.72)	2.56 (0.43)	11.74 (1.35)	2.83 (0.13)	299.46 (26.07)	92.98 (2.12)	-
**Total**	42.01 (1.88)	20.93 (1.48)	100	2.91 (0.14)	319.62 (8.47)	100	-

*^13^C and ^15^N values are in excess, i.e., the natural abundance control was subtracted. Standard errors are shown in brackets; n = 7*.

**Table 2:** ^13^C, C, and N in dissolved organic matter and microbial biomass. C_mic_, N_mic_, DOC, and TDN are means in μg g^−1^ dry weight, and ^13^C_mic_ and ^13^C in DOC are atom% excess.

**Pool**		**C_**mic**_**	**N_**mic**_**	**DOC**	**TDN**	**^**13**^C_**mic**_**	**^**13**^C in DOC**	**(C/N)_**mic**_**
Rhizosphere soil	no N	610.28 (15.95)	142.12 (4.69)	154.68 (6.27)	27.62 (3.16)	0.47^*^ (0.05)	0.17^*^ (0.06)	4.31 (0.10)
	+N	665.6 (24.63)	141.96 (5.94)	139.26 (3.79)	29.98 (7.4)	0.61^*^ (0.06)	0.15^*^ (0.06)	4.70 (0.12)
Bulk soil	no N	655.13 (30.75)	126.36 (6.05)	145.35 (25.47)	25.97 (3.3)	0.20^*^ (0.02)	0.03^*^ (0.01)	5.26 (0.34)
	+N	620.07 (18.81)	132.21 (7.75)	179.85 (11.92)	36.2 (7.86)	0.18^*^ (0.04)	0.03^*^ (0.01)	4.77 (0.24)
Litter	no N	1725.91 (137.64)	286.29 (33.68)	2867.67 (112.47)	811.58 (45.18)	0.10^*^ (0.05)	0.01^*^ (0.00)	6.22 (0.44)
	+N	1822.26 (206.47)	259.31 (39.96)	3004.41 (95.22)	857.49 (110.57)	0.09^*^ (0.03)	0.01^*^ (0.00)	7.53 (0.68)

*C_mic_, C in microbial biomass; ^13^C_mic_, ^13^C in microbial biomass; N_mic_, N in microbial biomass; DOC, dissolved organic C; TDN, total dissolved N; (C/N)_mic_, C/N ratio in microbial biomass. Standard error is shown in brackets. Asterisks indicate significant difference to control (p < 0.05) in atom% ^13^C (comparison via Mann-Whitney U-test for equal medians; n = 7). No significant differences were detected between +N/noN compartments (comparison via Mann-Whitney U-test for equal medians; n = 7)*.

Concurrently, 7% of the total added ^15^N were transferred from the litter compartment to the plant within 48 h (Table 1). Significant ^15^N enrichment was measured in stems and plant roots of the N-treated side, and a small but significant enrichment was found in roots of the untreated side (Figure 3B), which can be explained by plant-internal re-distribution of N. In contrast, ^15^N enrichment of bulk and rhizosphere soil on the N-treated side was very low [δ^15^N of 9.8 ± 2.4‰ and 17.7 ± 4.2‰, respectively (average ± SE)] compared to those of litter and roots (δ^15^N of 540.9 ± 45.2‰ and 393.5 ± 165.7‰, respectively). This demonstrates the effectiveness of the double-layer mesh containing an air-filled space in the middle as a diffusion barrier, and indicates that N was mainly transferred directly via fungal hyphae from the litter to the roots. Soil of the N untreated side was not enriched in ^15^N.

**Figure 5:**
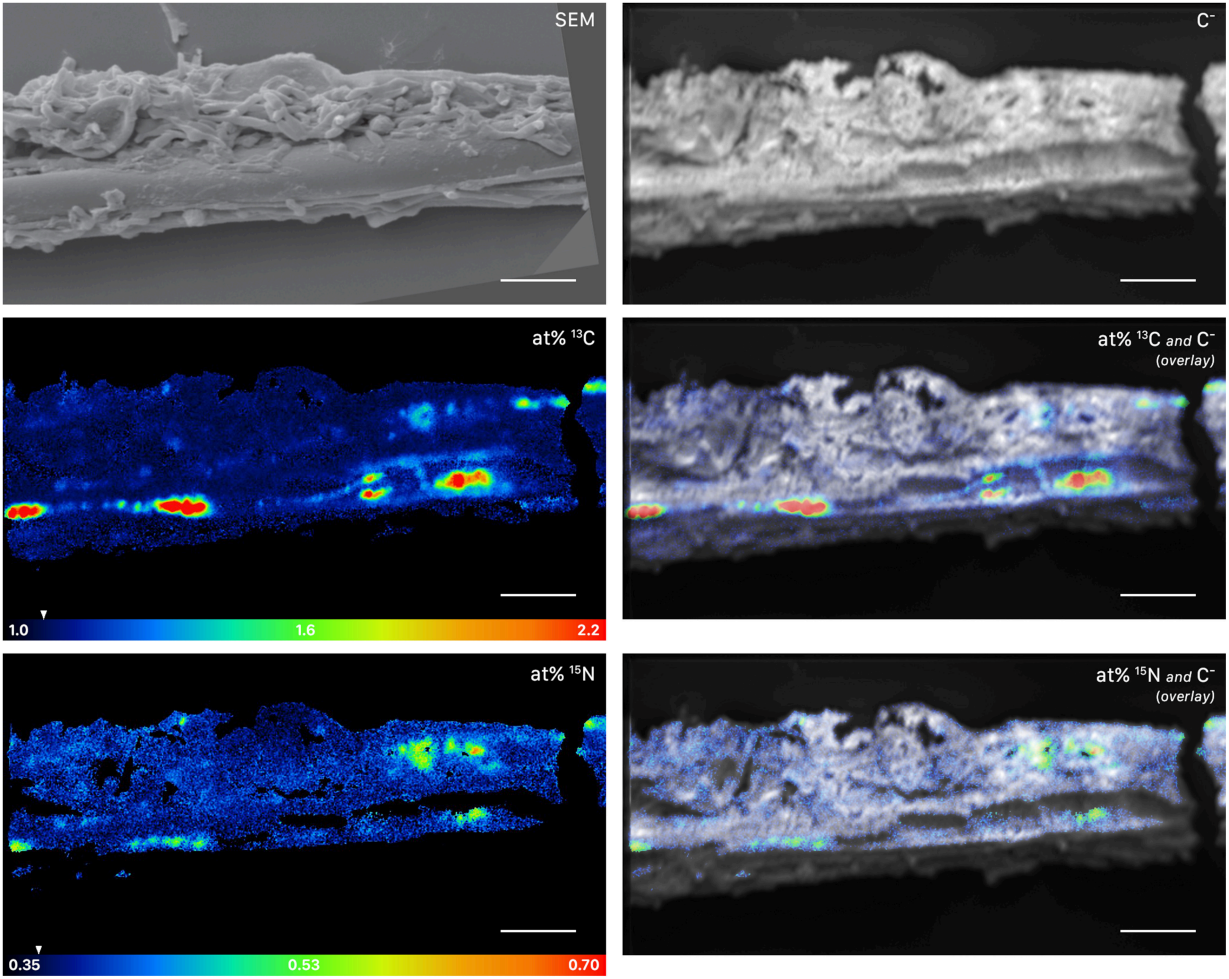
NanoSIMS and SEM images of a root-distant fungal hypha extracted from a litter compartment of a dual-labeled plant box, depicting ^13^C and ^15^N labeled regions within the hypha. The SEM image shows the morphology of the sample prior to NanoSIMS measurement. NanoSIMS images were acquired at an erosion depth below the hyphal surface, visualizing the presence of isotopically enriched compounds inside the hypha. Isotopic label contents are displayed as atom%. The white arrows at the color-scales indicate the natural isotopic abundance values, determined on the unlabeled control (1.08 atom% ^13^C and 0.37 atom% ^15^N). The upper limit of the atom% ^13^C scale is set to 2.2; however, maximum local values, extracted from individual cycles, range up to 3.6 atom% ^13^C. Secondary ion signal intensity thresholds were set to 62 and 60 counts/(sec^*^pixel) for ^12^C^13^C^−^ and ^12^C^15^N^−^, respectively. SEM, scanning electron microscopy image (secondary electrons); C^−^, accumulated ^12^C^−^ and ^13^C^−^ secondary ion signal intensity distribution images, indicating the morphology of the sample during NanoSIMS analysis; at% ^13^C, carbon isotope composition image; at% ^15^N, nitrogen isotope composition image. Overlay images are composites of the C^−^ and isotope images. NanoSIMS images consist of accumulated z-stacks obtained from 19 consecutive scans (displayed in Animation S1). Scale bars, 5 μm.

**Figure 6:**
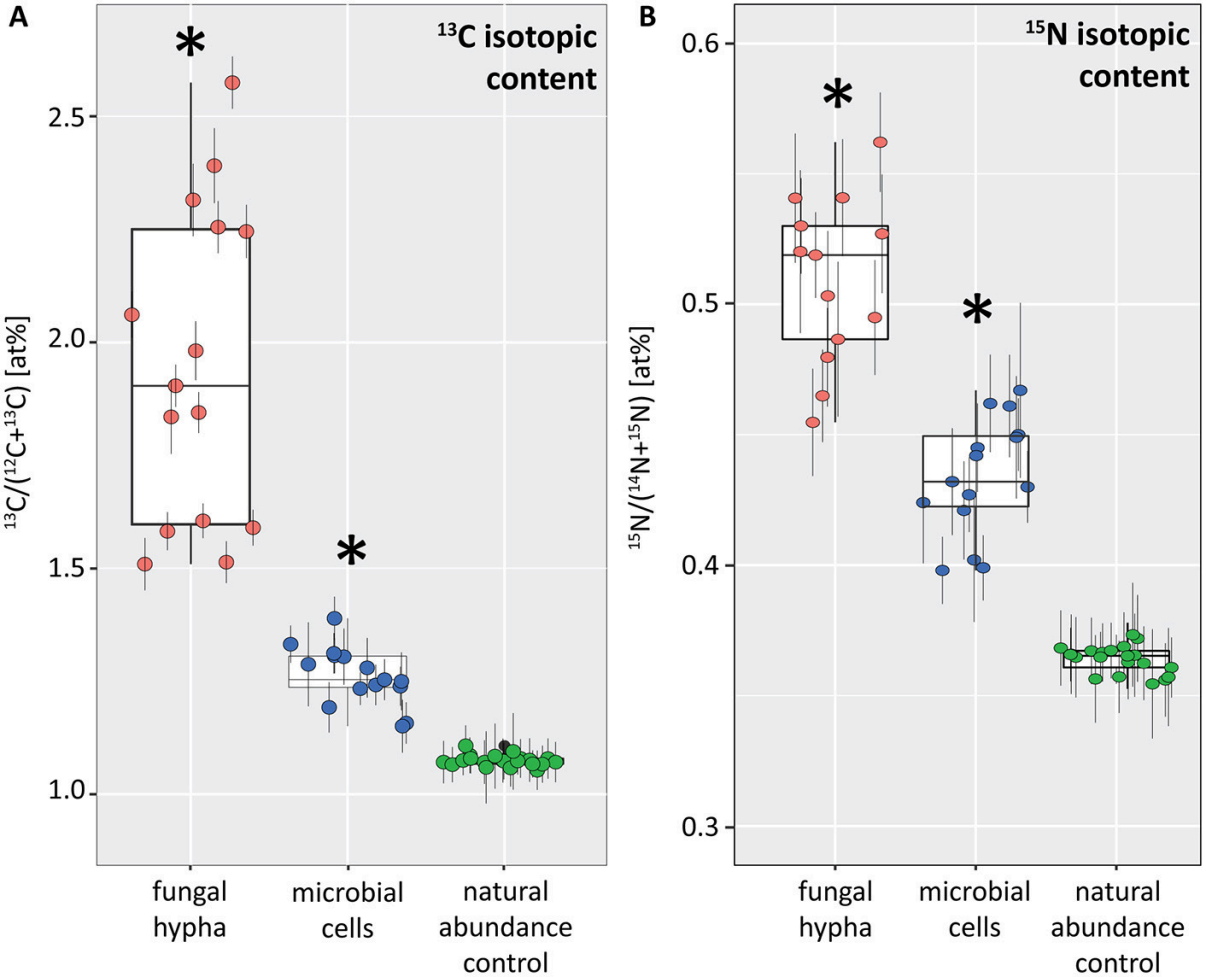
Boxplots depicting the ^13^C **(A)** and ^15^N **(B)** isotopic content (in atom%) for a fungal hypha (displayed in Figure 5) and hyphosphere microorganisms (displayed in Figure 7) from dual-labeled plant-boxes, along with the values obtained from measurement on the natural abundance control. Each data point represents an individual region of interest (ROI) extracted from NanoSIMS images depicted in Figures 5, 7, error bars refer to the estimated analytical uncertainty (1σ) due to counting statistics (see section Materials and Methods). Significant levels of isotopic enrichment (*p* < 0.001) are indicated with an asterisk (^*^). Data for the natural abundance control were obtained from microbial cells associated to the surface of a hypha extracted from a non-labeled control box. With respect to the values of the fungal hypha it should be noted that these refer to the averages over 19 consecutive scans. Owing to the observed variation of enrichment within the z-stack (see Animation S1), the displayed maxima rather represent conservative estimates.

This symbiotic, bi-directional transport of recent photosynthates and added N was confirmed by NanoSIMS investigation of a fungal hypha retrieved from the litter compartment. The measurement was conducted at a depth below the hyphal surface, illustrating the incorporation of C and N isotopes inside the hypha (Figure 5, Animation S1). A carry-over of ^13^C and ^15^N atoms through the NanoSIMS measurement process (e.g., by atomic mixing) from surface-associated microbial cells can be ruled out, as overlay of the isotope composition images with the SEM image shows that regions of high isotopic enrichment within the lumen correspond to areas on the hyphal surface that were devoid of microbial cells (Figure S4). Region of interest (ROI) specific data evaluation of NanoSIMS images revealed that the fungal hypha was significantly enriched in both ^13^C and ^15^N (*p* < 0.001) compared to the natural abundance control. The isotopic signature of individual ROIs ranged from 1.5 to 2.6 atom% ^13^C and from 0.46 to 0.56 atom% ^15^N (Figure 6).

### Transfer of Photoassimilated Carbon to Soil Microbes via Mycorrhizal Hyphae

Our results demonstrate a transfer of recently photoassimilated C to bacteria in both the rhizosphere and the hyphosphere. The sum of bacteria-specific PLFAs as well as specific bacterial groups (Gram-positive, Gram-negative, Actinobacteria) were significantly enriched in ^13^C in the litter compartment and the rhizosphere soil compared to natural abundance controls (Mann-Whitney *U*-test, *p* < 0.05), which is illustrated by positive atom% excess values in Figure 4. In bulk soil, in contrast, only Gram-positive bacterial PLFAs were significantly enriched (Figure 4, Table S2). All individual PLFA biomarkers were significantly enriched in atom% ^13^C in the litter compartment of the side that did not receive N (Table S2). We found no preferential ^13^C transfer to specific bacterial groups, although the actinobacterial PLFA was slightly less enriched compared to other Gram-positive bacterial PLFAs, and Gram-negative bacterial PLFAs. All bacterial groups were similarly ^13^C enriched in all pools (in atom% ^13^C) except for Gram-positive bacteria which were more enriched in litter compared to bulk soil (Figure 4). Furthermore, dissolved organic carbon (DOC) was significantly enriched in ^13^C in the litter compartment, in addition to bulk and rhizosphere soil (Table 2).

**Figure 7:**
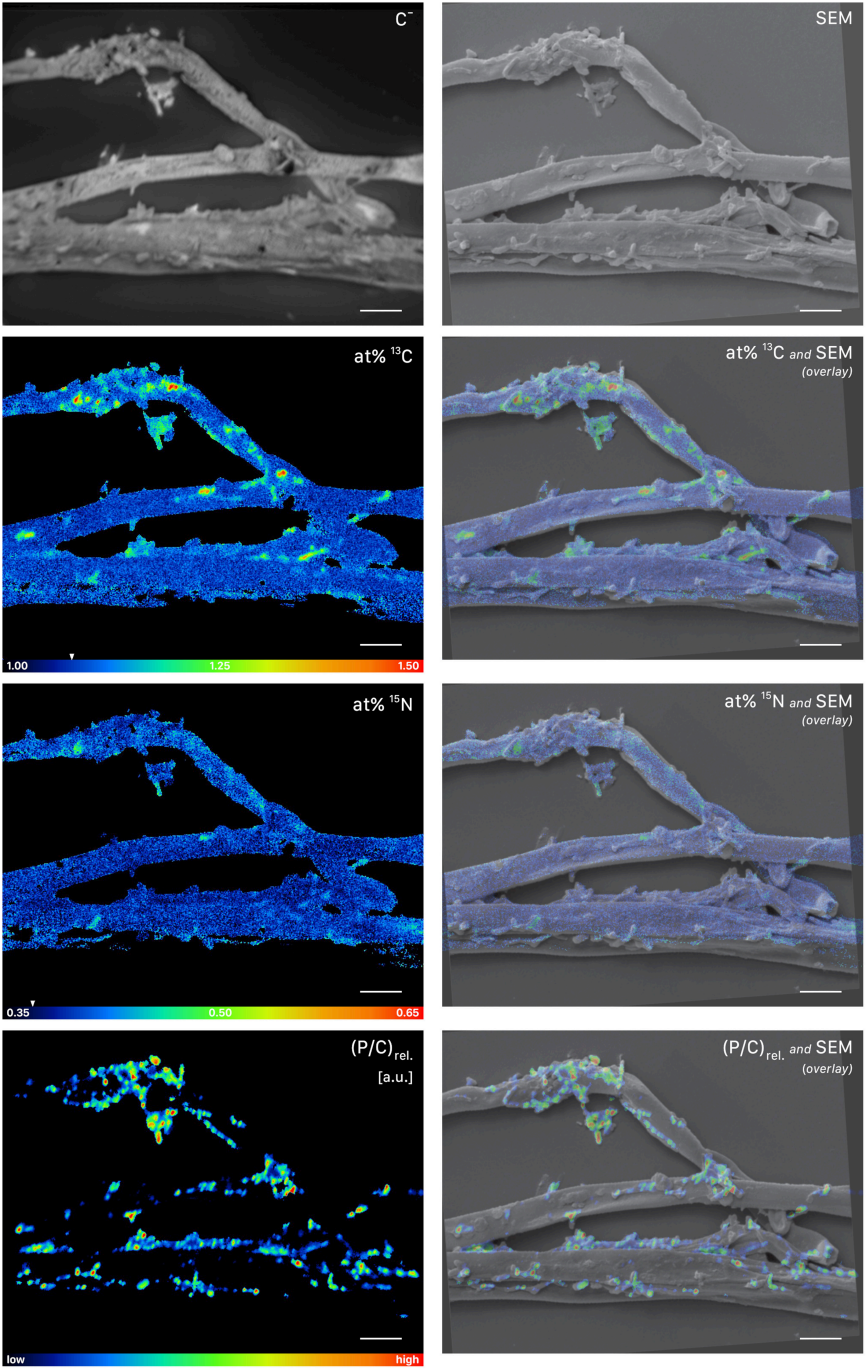
NanoSIMS and SEM imaging of cells attached to root-distant fungal hyphae extracted from a litter compartment of a dual-labeled (^13^C and ^15^N) plant-box. SEM, scanning electron microscopy image (secondary electrons); C^−^, accumulated ^12^C^−^ and ^13^C^−^ secondary ion signal intensity distribution images, indicating the morphology of the sample during NanoSIMS analysis; at% ^13^C, carbon isotope composition image, showing ^13^C enrichment in microbial cells on the surface of the hyphae; at% ^15^N, nitrogen isotope composition image. Isotopic label contents are displayed as atom%. The white arrows at the color-scales indicate the natural isotopic abundance values, determined on the unlabeled control (1.08 atom% ^13^C and 0.37 atom% ^15^N). The upper limit of the atom% ^13^C scale is set to 1.50, maximum values range up to 1.56 atom%. Secondary ion signal intensity thresholds were set to 119, 51 and 58 counts/(sec^*^pixel) for ^12^C^13^C^−^, ^12^C^15^N^−^, and ^31^P^−^ respectively. (P/C)_rel._, relative phosphor-to-carbon elemental ratio image as inferred from C^−^ normalized ^31^P^−^ secondary ion signal intensities, indicating the presence of microbial cells on hyphal surfaces. Overlay images are composites of the SEM and NanoSIMS images. NanoSIMS images consist of accumulated z-stacks obtained from 24 consecutive scans. Scale bars, 5 μm.

NanoSIMS imaging revealed a spatial pattern of ^13^C and ^15^N hotspots on the surface of the fungal hyphae collected from the litter compartment resembling the size and distribution of microbial cells. Chemical composition (such as the NanoSIMS-visualized relative contents of phosphor and nitrogen) and structural information (provided by SEM imaging and the NanoSIMS C^−^ secondary signal intensity distribution) supported their identification as hyphosphere microbial cells (Figure 7, Figure S3). These microbial cells were significantly enriched in ^13^C and ^15^N (*p* < 0.001) compared to a natural abundance control, with ROI specific isotopic contents ranging from 1.2 to 1.4 atom% ^13^C and from 0.40 to 0.47 atom% ^15^N (Figure 6).

### Effect of Nitrogen Addition on Soil Microbes and Belowground Carbon Flux

#### Microbial Reaction to Nitrogen Addition

**Figure 8:**
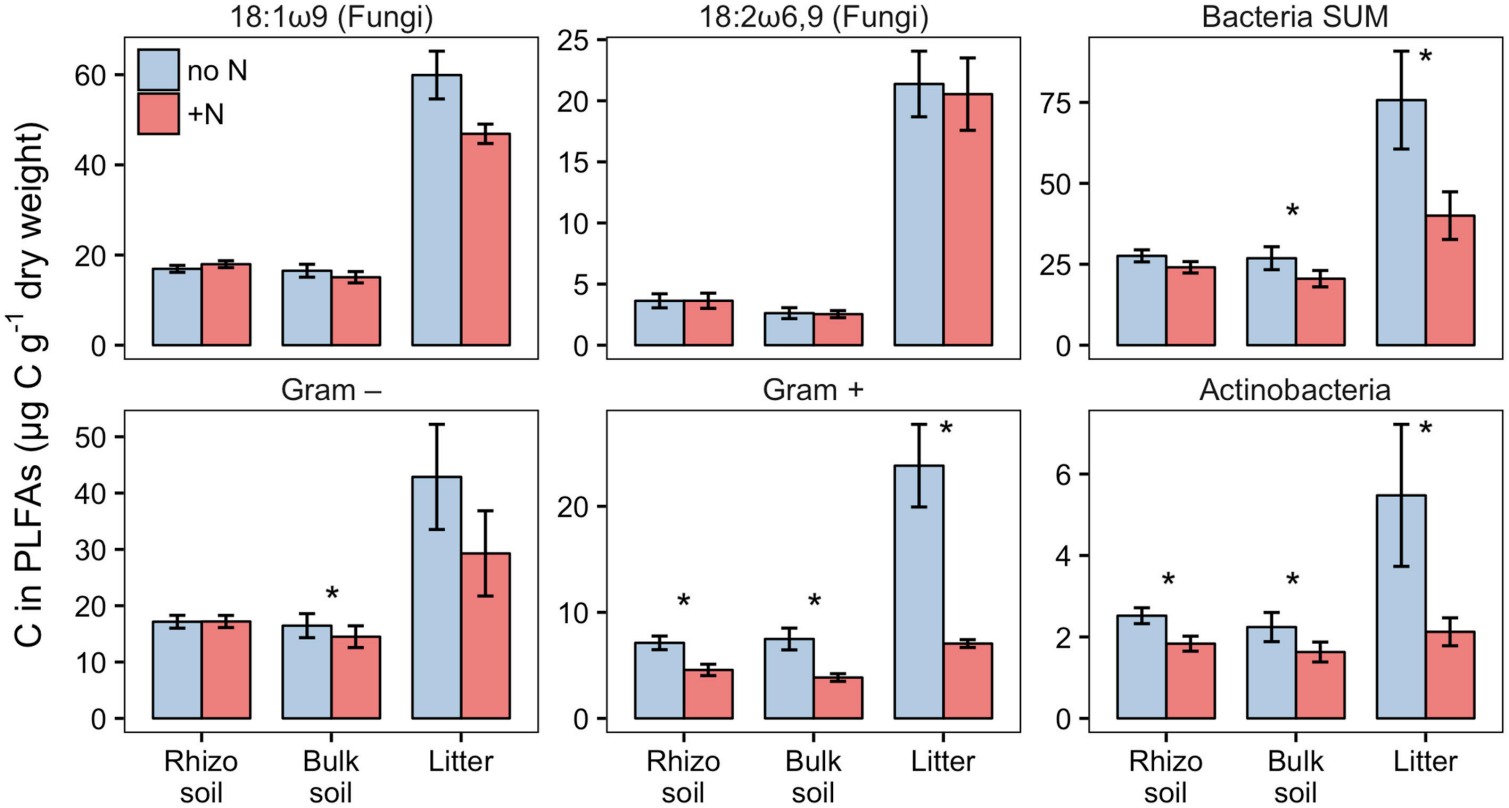
Biomass of fungi and bacterial groups in rhizosphere, bulk soil and litter pools of N-treated and untreated sides of dual-labeled (^13^C and ^15^N) plant boxes, represented by PLFA biomarkers. Biomass of Actinobacteria and Gram-positive bacteria (excluding the Actinobacterial PLFA) declined significantly in both litter and soil compartments with N addition (i.e., to the litter compartment). Significant differences between N-treated (+N) and untreated side (no N) are indicated with asterisks (Mann-Whitney *U*-test for paired samples; *p* < 0.05; *n* = 6). Although not statistically significant, the adverse effect of N addition is also present in Gram-negative bacteria in litter, and the fungal PLFA 18:1ω9 in litter and bulk soil (*p* < 0.01). Error bars represent the standard error. Differences between soil and litter pools of the untreated side were analyzed with the Kruskal-Wallis rank sum test. Significant test results (*p* < 0.05) were followed by Dunn's *post-hoc* test of multiple comparisons with Bonferroni correction (adj. *p* < 0.05). Rhizosphere soil and litter, as well as bulk soil and litter, were significantly different in all groups except for Actinobacteria (not shown).

**Figure 9:**
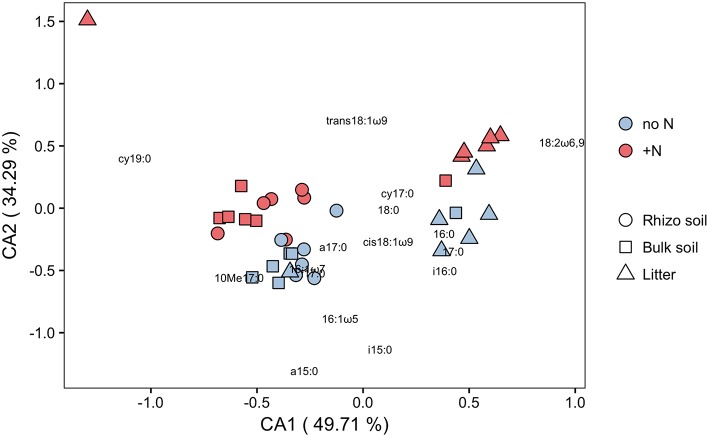
Correspondence analysis (CA) of C in PLFAs (μg C g^−1^ dry weight) in N-treated and untreated compartments of dual-labeled (^13^C and ^15^N) plant boxes. Close distances between individual PLFAs (depicted as text) and soil/litter-pools of individual split-root boxes (depicted as symbols) indicate higher abundance of respective PLFAs in concerned pools. Axes notations give the proportion of variance explained on each coordinate in percent. Colors refer to box-sides with untreated (no N) and N-treated (+N) litter compartments. ANOSIM analysis shows significant difference between pools (*R* = 0.566, *p* = 0.001) as well as between N-treatments (*R* = 0.103, *p* = 0.011). This analysis indicates (i) a distinctly different microbial community between soil pools (rhizosphere soil, bulk soil) and litter, and (ii) that N addition affects not only community structure in hyphae-only litter compartments, but also in soil compartments (cf. Figure 8).

PLFA analysis showed that several microbial groups significantly declined in response to N addition (Figure 8, Table S3). This effect of N was particularly pronounced in Actinobacteria and Gram-positive bacteria in general, but also Gram-negative bacteria and the fungal PLFA 18:1ω9 slightly declined. Surprisingly, the effect on Gram-positive bacteria was not restricted to the litter compartment to which N was added, but also occurred in the bulk soil and rhizosphere of the adjacent soil compartments (Figure 8). In line with this, correspondence analysis of PLFAs showed that microbial community composition differed not only between litter and soil, but also changed in response to N addition (Figure 9). Again, N addition resulted in a shift in community structure not only in the litter compartment to which N was added, but also in the adjacent soil compartment.

To rule out that this difference in microbial communities was caused by a systematic discrepancy between the compartments of the split-root boxes, we analyzed PLFAs in both litter compartments of plant boxes that did not receive N at all (*n* = 4, data not shown). We found no difference between box sides in these plant boxes, nor in boxes without plants, which also did not receive a N treatment (*n* = 3, data not shown).

In contrast to the PLFAs, microbial biomass C based on CFE was unaffected by N addition (Table 2).

#### Nitrogen Availability Affects Carbon Transfer to Soil Microbes

**Figure 10:**
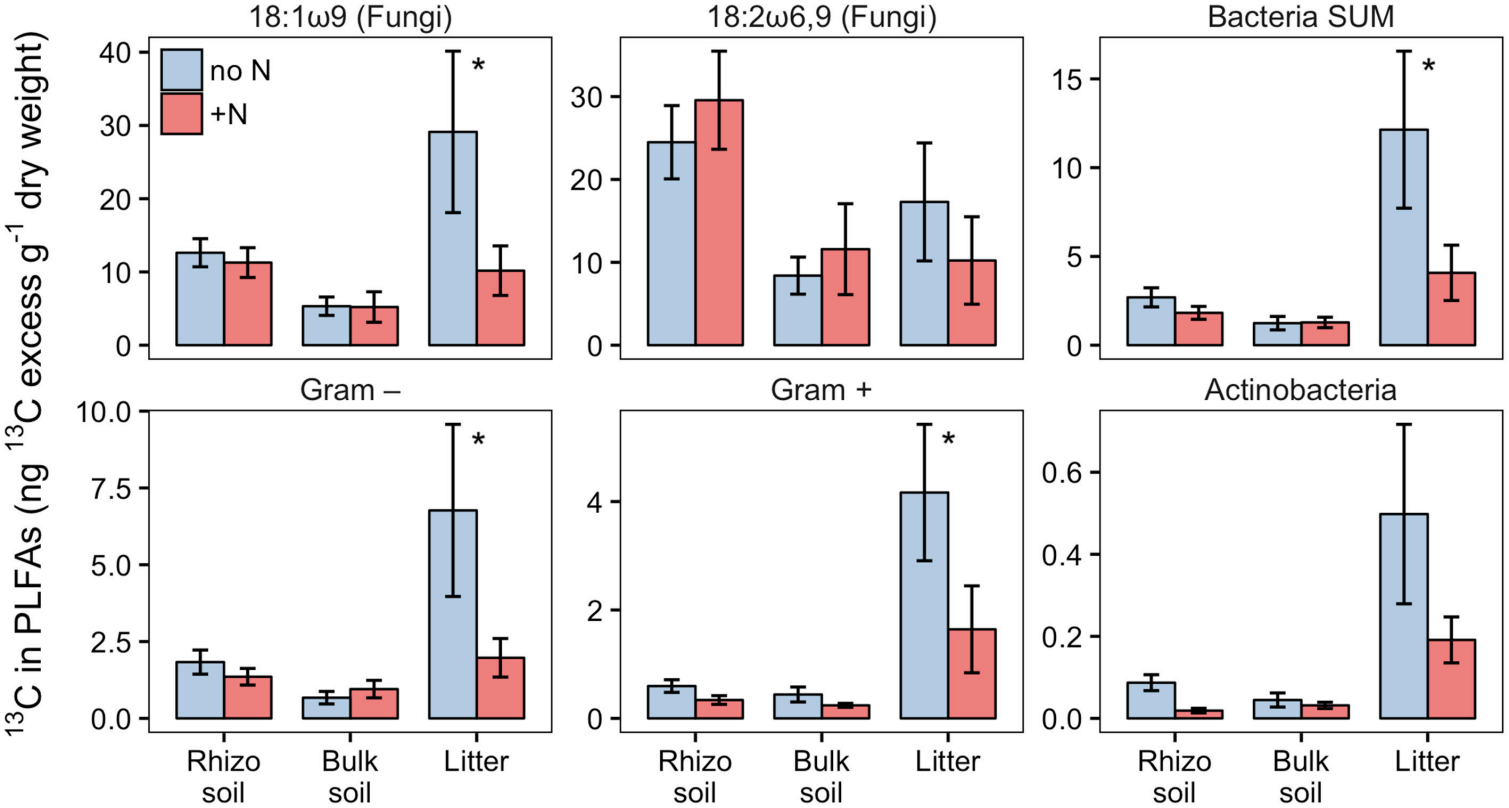
^13^C-excess in PLFAs per g dry weight in N-treated and untreated compartments of dual-labeled (^13^C and ^15^N) plant boxes. Enrichment of bacteria-specific PLFAs in the litter compartment indicates allocation of photoassimilated ^13^C via ECM hyphae to root-distant bacteria. Significant differences between untreated (no N) and N-treated side (+N) are indicated with asterisks (Mann-Whitney *U*-test for paired samples; *p* > 0.05; *n* = 6). Error bars represent the standard error.

Based on PLFA analysis, N addition to the litter compartment significantly decreased ^13^C transfer to Actinobacteria and other Gram-positive bacteria, on a per g dry weight basis (Figure 10). This is caused by a reduction in biomass of the respective groups (Figure 8) combined with a constant or decreased relative enrichment of ^13^C (Figure S5, Table S2). This indicates that, in addition to a loss of biomass, N treatment also affected the transfer of recent photosynthates to hyphosphere bacteria.

Relative isotopic enrichment (atom% excess ^13^C) in PLFAs specific for bacteria and the fungal-specific PLFA 18:1ω9 tended to decrease (although not significantly) in litter after N addition (Figure S5). However, correspondence analysis shows no effect of N addition on relative ^13^C enrichment (Figure S6). Moreover, we did not observe a significant effect of N addition on relative ^13^C enrichment in microbial biomass based on CFE (Table 2).

## Discussion

The release of recent plant photosynthates into the soil has been shown to accelerate microbial decomposition of soil organic matter (Kuzyakov et al., 2000; Cheng et al., 2014), which has been identified as a strong driver for terrestrial C and nutrient cycling (Heimann and Reichstein, 2008). While research has traditionally focused on the effect of direct root exudation, recent studies highlight the contribution of an alternative “mycorrhizal pathway,” demonstrating a transfer of plant-derived C via AM mycorrhizal fungi to soil bacteria (Toljander et al., 2007; Drigo et al., 2010; Cheng et al., 2012; Kaiser et al., 2015; Paterson et al., 2016). Very little, however, is known about a possible transfer of recent photosynthates to soil via ECM fungi, and interactions of ECM fungi with soil bacteria. Here, we provide evidence that plant-photoassimilated C is rapidly transferred to root-distant soil bacteria via ECM hyphae, indicating that ECM fungi readily share this resource with soil saprotrophs. Contrary to our hypothesis, however, the addition of labile N did not result in increased hyphal C transfer to bacteria. Instead, bacterial biomass and its incorporation of recent photosynthates declined with N addition not only in the litter compartment to which N was added, but also in adjacent untreated soil compartments, indicating a complex response of fungal-bacterial interactions to changing N availabilities.

### Ectomycorrhizal Fungi Transfer Recent Photosynthates to Root-Distant Areas

Recent photosynthates were transferred to root-inaccessible areas in our system within hours. This transport of plant-derived C into litter compartments was most likely restricted to fungal hyphae, as our experimental design prevented diffusion and penetration of roots into the litter compartment by means of a double-layer mesh with an air-filled gap that could only be penetrated by hyphae. Hyphal transport of recently photoassimilated C is supported by significant ^13^C-enrichment in microbial biomass based on CFE (Table 2) as well as fungal- and bacterial-specific PLFAs in the litter compartment (Figure 4, Table S2). Furthermore, NanoSIMS imaging provided evidence of significant ^13^C enrichment inside a fungal hypha obtained from the litter compartment (Figure 5).

The similarity of ECM fungal communities in rhizosphere and litter compartments, together with the near absence of ECM fungal communities in litter compartments of unplanted boxes (Figure 2), strongly indicates a vital connection between plant roots and associated litter compartments by mycorrhizal hyphae. While some transfer of recently plant-assimilated ^13^C to the litter compartment via saprotrophic fungi cannot be ruled out, it is considered to be negligible because of the high degree of mycorrhization (which likely prevents high rates of root exudation) and the short time scales considered. Saprotrophic fungi are known to use complex organic matter as their main C source, rendering them unlikely to introduce substantial amounts of labile root exudates into the litter compartment within short time scales. We therefore assume that C transport via ECM hyphae accounted for the largest fraction of ^13^C allocated to the litter compartment. In particular, the interior of the hypha investigated by NanoSIMS was highly enriched in ^13^C (up to 2.6 atom% excess ^13^C, Figure 5), clearly exceeding ^13^C enrichment of the microbial biomass in the rhizosphere (up to 0.74 atom% excess ^13^C), and also the ^13^C enrichment of fine roots (0.88 atom% excess ^13^C), with the latter representing a potential source of C for saprotrophic fungi. While it is impossible to identify its phylogenetic identity, this strongly indicates that this hypha was mycorrhizal, as other, not plant-associated fungi would not be able to acquire that much plant-photoassimilated C in such a short time.

### Soil Bacteria Receive Recent Photosynthates From Ectomycorrhizal Fungi

Providing free-living saprotrophs with low-molecular-weight C compounds to supplement their energy demand is a potentially viable strategy for ECM fungi to accelerate decomposition of recalcitrant organic matter. We provide evidence for a direct, short-term transfer of recent photosynthates (i.e., shorter than 29 h) via fungal hyphae to hyphosphere bacteria. This is supported by significant ^13^C-enrichment not only of (a) bacteria-specific PLFAs in a compartment only accessible by fungal hyphae (Figures 4, 10), but also (b) hyphae-attached microbial cells as visualized by NanoSIMS (Figure 7). Together with the fact that DOC was significantly ^13^C-enriched, this indicates consumption of ^13^C-enriched exudates by hyphae-associated microbes and incorporation of this C into cellular biomass. This study is the first one visualizing the incorporation of stable isotope labeled plant-derived C from ECM fungal hyphae into hyphae-associated microorganisms.

Another explanation for the ^13^C enrichment of bacteria in litter compartments could be the acquisition of ^13^C by saprotrophic microbes feeding on ECM hyphal necromass. This appears however likely negligible within the short period between the onset of ^13^C-CO_2_ pulse labeling and harvest, since the turnover rate estimates for ECM hyphae in natural systems range from weeks to several months (Ekblad et al., 2013).

Unexpectedly, there was no preference in C transfer to specific bacterial groups, neither in the rhizosphere nor in the litter compartment as far as the resolution of the PLFA method allowed to trace this. This somewhat contrasts previous studies in which Gram-negative bacteria preferentially utilized plant-derived compounds in the rhizosphere (Kramer and Gleixner, 2006, 2008; Esperschütz et al., 2009; Pickles et al., 2016).

Interestingly, the relative ^13^C enrichment of bacterial PLFA biomarkers was as high, or even higher in the litter compartment than in rhizosphere or bulk soil, while relative ^13^C enrichment of the fungal biomarker 18:2w6,9 strongly decreased from rhizosphere soil to the litter compartment. Furthermore, bacteria incorporated the highest absolute amounts of ^13^C in the litter compartment (untreated side in Figure 10). Together, this suggests higher hyphal C exudation in litter compared to soil compartments, indicating that bacteria received more C via the mycorrhizal hyphal pathway in the litter compartment than via combined root and mycorrhizal hyphal exudation in the rhizosphere. ECM fungal hyphae are known to increase branching and growth when they reach organic-rich substrates (Agerer, 2001, 2006), which may have happened in our litter compartments. Such a growth pattern would tremendously increase the mycelial surface area and number of hyphal tips (Katz et al., 1972), which could, particularly under the assumption that ECM hyphal exudation concentrates on hyphal tips (Unestam and Sun, 1995; Sun et al., 1999), explain the higher total rate of ^13^C transfer to bacteria that we observed in the litter compartments.

Evidence of hyphal C translocation to bacteria is missing in ECM systems and is scarce even for AM systems, where it is most often based on DNA and PLFA stable isotope probing (SIP) methods, which lack a spatial context. Here, we demonstrate the transfer of labeled compounds from fungi to soil microbes in an ECM system by a spatially explicit approach, which provides insight into the local aspects of this interaction, and allows the *in situ* visualization of the stable isotope composition of microbial cells associated with fungal hyphae via NanoSIMS analysis. While NanoSIMS represents a promising topochemical analysis technique to study C and nutrient transfer in fungal-bacterial interactions, it has only been used in a few studies for this purpose so far. Worrich et al. (2017), for example, investigated fungal-bacterial interactions in a synthetic microbial ecosystem, demonstrating a direct transfer of water and nutrients from hyphae of the oomycete *Phytium ultimum* to bacterial cells of *Bacillus subtilis*. In a pioneering effort of visualizing microscale rhizosphere C flow through an undisturbed plant-soil system, Vidal et al. (2018) repeatedly exposed wheat, an AM plant, to ^13^C-CO_2_ over a time period of 10 weeks and subsequently analyzed the distribution of ^13^C in undisturbed rhizosphere samples using NanoSIMS. They found, amongst other results, a ^13^C enriched spot, presumably a bacterial cell, on a fungal hypha associated with a wheat root. However, due to the long-term labeling approach, this finding can't be attributed to a hyphal transfer of recently assimilated plant C to microbial cells (which was also not the aim of that study), but may reflect accumulation of plant-derived organic C in soil bacteria over various pathways and longer time scales.

### Relationship Between Ectomycorrhizal Hyphae and Associated Bacteria

The addition of ^15^N-labeled labile N compounds affected microbial community structure within days, causing a loss of biomass of certain bacterial groups. Actinobacteria and other Gram-positive bacteria declined strongest (*p* < 0.05), but Gram-negative bacteria and the fungal PLFA 18:1ω9 tended to decline as well (*p* < 0.1; Figure 8). Only the fungal PLFA 18:2ω6,9 remained unaffected by the treatment. The two fungal-specific PLFA biomarkers also differed in their absolute abundance and ^13^C incorporation: while 18:1ω9 was more abundant than 18:2ω6,9 (Figure 8), it was relatively less enriched in ^13^C (Figure 4). Together with their different response to N addition, this could indicate that in our system 18:1ω9 is more indicative of fungi in general, including free living saprotrophs, while 18:2ω6,9 could be more specific for ECM fungi.

In contrast to the PLFAs, microbial biomass C based on CFE was unaffected by N addition (Table 2). Potential reasons for this could be (i) the higher precision of PLFA analysis compared to CFE or (ii) differences in the target of the two methods. Phospholipids mainly occur in cell membranes, thus emphasizing cell surface areas in PLFA measurements, while CFE lyses cells with the lysate being proportional to cell volumes. For example, if a deteriorating effect on microbial biomass affected small microbial cells (e.g., small bacteria) more than larger cells (e.g., fungi or larger bacteria), surface areas (PLFAs) would decrease stronger than volume (CFE-based biomass). This possibility is indirectly supported by PLFA analysis which also indicated a community shift in response to N addition.

The strong and very rapid negative effect of labile N on bacterial biomass seems counter-intuitive, as we would rather expect a positive effect of N on bacterial growth. The amount of N we have added (0.168 mg NH_4_-N, and 0.168 mg N as amino acids) makes up for around 5% of the total dissolved N in the litter compartment, representing a moderate increase in the concentration of labile N compounds. However, the negative effect on microbial biomass was not restricted to the litter compartments, which received labile N inputs, but it also significantly affected the soil compartments separated from them by the hyphal-penetrable mesh.

Our ^15^N measurements indicate that around 10% of the added N was transferred from the litter to the soil in total (Table 1), which would yield an enhancement of total dissolved N in this compartment by 0.5% (data not shown). As this represents only a negligible increase, we can rule out a direct effect of enhanced N availability on microbial biomass and community structure. As the effect however clearly took place not only in the litter, but also in the adjacent soil compartments, we can only speculate that it must have been caused by a systemic reaction of the fungi or the plant to altered local availability of N. If it was a fungal reaction it would be restricted to those fungi growing in the N-treated side of the box, i.e., those which connect the N-treated litter compartment with the plant roots in the mesh-separated soil compartment. If it was a plant reaction, it needed to have been directed toward the part of the root system that received more N.

One possible mechanism could be that when ECM fungi were exposed to easily available N in excess, their interaction with associated bacteria drastically changed from cooperative to competitive. As a consequence, ECM fungi may have taken up the main part of the added ^15^N, transferring it toward plant roots, while impairing bacterial growth along the whole hyphal “supply line” (i.e., not only in the litter compartments, but also in the adjacent soil compartments). The latter could have happened in different ways, for example by reducing the input of labile C, which may be vital for parts of the bacterial community, or by actively employing allelopathic strategies, such as the production of antibiotics.

In fact, fungi are known to produce a plethora of bacterial and fungal antibiotics (Keller et al., 2005), and to use them in competition against saprotrophic fungi (Fernandez and Kennedy, 2015). Furthermore, bacteria of the genus *Streptomyces* (Actinobacteria), which is the largest antibiotic-producing bacterial genus known (Watve et al., 2001) have been found to live in close association with ECM fungi (Schrey et al., 2005; Seipke et al., 2012). ECM fungi thus seem to have the potential to take allelopathic actions against bacterial saprotrophs when they switch to a competitive situation. To test if this actually happens goes however beyond the scope of this study and warrants further research.

As another option, ECM fungi may have reduced the transfer of plant-derived C to their bacterial competitors. This is supported by our results, which show a significant decline in the absolute amount of ^13^C incorporated into bacterial PLFAs and the fungal PLFA 18:1ω9 after N addition (Figure 10). As bacterial and fungal PLFA biomarkers received significantly lower amounts of ^13^C, but ^13^C enrichment in DOC was unchanged, we conclude that in total less ^13^C was transferred via mycorrhizal hyphae to the litter compartment after addition of N (Table 2). An analogous reaction is often observed in plants, where C allocation to the rhizosphere decreases with N fertilization (Kuzyakov and Domanski, 2000). The question remains whether a decrease in labile C input could have led to such a rapid decline in bacterial biomass, which seems unlikely to be caused just by starvation of bacterial cells. The hyphosphere, however, may be—similar to the rhizosphere—a microbial “hotspot,” i.e., a spatially restricted soil volume with exceptionally high abundances and turnover rates of microbes, caused by high rates of labile C input (Kuzyakov and Blagodatskaya, 2015). High turnover rates are defined as fast growth rates balanced by high mortality rates. In an environment not limited by substrate availability, mortality is likely driven by density dependent mechanisms, such as predation, virus activity, or negative bacterial interactions (West et al., 2007; Ratzke et al., 2018). It is thus possible that diminishing substrate input decreases bacterial growth rates, but mortality rates may stay high until population density has substantially decreased, which may also explain the rapid decline in microbial biomass.

While our results indicate that N availability affects the relationship of mycorrhizal fungi and soil microbes, it has to be kept in mind that we added only easily available forms of N, and only at a single concentration. The system may have responded in a different way if other quantities or qualities of N, such as complex organic N, were added. While this would be interesting to investigate, it goes beyond the scope of this study.

## Conclusions

Our results show a transfer of recent photosynthates via ECM hyphae to associated soil microbes in root-inaccessible areas within hours, supporting the hypothesis of priming in an ECM hyphosphere as a potential mechanism to increase access to nutrients from soil organic matter for ECM fungi and their host plants.

Contrary to our expectations, elevated local availability of labile N compounds did not result in higher hyphal exudation rates. Instead, we found a strong decline in biomass of Actinobacteria and other Gram-positive bacteria in response to N addition. This decline seems to have been caused by ECM fungi acting on soil bacteria, as the effect was observed in soil compartments where fertilized N would only be available for bacteria through hyphal transport.

Based on our observations, we suggest the relation between ECM hyphae and soil bacteria to be on a “razors edge,” tilting between mutualistic and antagonistic interactions depending on environmental conditions. It is likely that fungi occupy the more sustained position in such an opportunistic mutualism, by controlling C flow to bacteria. Accordingly, hyphosphere priming may be a highly controlled process with hyphal exudation being rapidly adjusted to changing soil nutrient availabilities.

## Author Contributions

CK conceived and coordinated the study. CK, AR, and DW designed the experiment. WM, RG, JW, VM, QZ, JP, MD, and SG did the experimental work, supervised by CK, PS, AR, and DW. JW, VM, SG and BI did the PLFA analysis. MD did the hyphal sample preparation, SEM analyses and ITS sequencing and analysis under supervision of MWe, SE, and DW. AS performed the NanoSIMS analysis in collaboration with MD and DW. MWa contributed to NanoSIMS method development. SG and SE analyzed the data. SG wrote the manuscript in close collaboration with CK and AS, with contribution of all co-authors.

### Conflict of Interest Statement

The authors declare that the research was conducted in the absence of any commercial or financial relationships that could be construed as a potential conflict of interest.
