# Supplementary material for: Rapid Transfer of Plant Photosynthates to Soil Bacteria via Ectomycorrhizal Hyphae and Its Interaction With Nitrogen Availability
Source: Front Microbiol. 2019 Feb 26;10:168. doi: 10.3389/fmicb.2019.00168 (PMC6399413; doi:10.3389/fmicb.2019.00168)
Supplement: Supplementary file 1 [file Table_1.DOCX]

Supplementary Tables

**Table S1** Comparison of total root dry mass of ^13^C- and ^15^N-labelled plants between the two soil compartments (corresponding to untreated (no N) and N-treated (+N) litter compartments). The ‘split-root’ setup did not result in a completely even distribution of root biomass between the two soil compartments. Box 17 was omitted in all of the analysis.

| **Plant box** |  | **no N** | **+N** | **Ratio (no N)/(+N)** |
| --- | --- | --- | --- | --- |
| **(sample #)** |  | **(g dry mass)** | **(g dry mass)** |  |
| 4 |  | 0.82 | 2.65 | 0.31 |
| 8 |  | 0.82 | 1.26 | 0.65 |
| 14 |  | 1.19 | 1.57 | 0.76 |
| 16 |  | 1.37 | 0.22 | 6.33 |
| 17 |  | 2.03 | 0.05 | 42.23 |
| 18 |  | 1.00 | 1.74 | 0.57 |
| 28 |  | 0.29 | 0.90 | 0.32 |
| 29 |  | 0.88 | 0.22 | 4.00 |
|  |  |  |  |  |

**Table S2** Atom% excess ^13^C in PLFAs. Values are means, standard error is shown in brackets (n=6). Asterisks indicate significant difference to control (p < 0.05; Mann-Whitney U test) in atom% ^13^C. Bold text indicates significant difference between untreated (no N) and N-amended (+N) side (p < 0.05; Mann-Whitney U test for paired samples).

|  |  | **Rhizosphere soil** | | **Bulk soil** | | **Litter** | |
| --- | --- | --- | --- | --- | --- | --- | --- |
| Phylogenetic specificity | PLFA | no N | +N | no N | +N | no N | +N |
| Fungi | 18.1ω9cis | 0.05*  (0.008) | 0.039*  (0.008) | 0.022*  (0.005) | 0.018*  (0.004) | 0.077* (0.04) | 0.03* (0) |
|  | 18.1ω9trans | 0.124*  (0.021) | 0.113*  (0.02) | 0.045  (0.01) | 0.058  (0.026) | 0.029* (0.011) | 0.017* (0.008) |
|  | 18.2ω6.9 | 0.692*  (0.094) | 0.792*  (0.074) | 0.38  (0.095) | 0.473  (0.188) | 0.114* (0.06) | 0.061* (0.027) |
| Bacteria general | 17.0 | 0.025*  (0.006) | 0.029  (0.008) | 0.011  (0.003) | 0.01  (0.002) | 0.022* (0.008) | 0.017* (0) |
| Gram negative bacteria | 16.1ω5 | 0.01*  (0.002) | 0.004  (0.002) | **0.004**  **(0.002)** | **0.006**  **(0.001)** | 0.034* (0.028) | 0.009* (0) |
|  | 16.1ω7 | 0.018*  (0.005) | 0.011*  (0.003) | 0.007  (0.003) | 0.006  (0.002) | **0.038* (0.025)** | **0.011* (0.003)** |
|  | cy17.0 | 0.006*  (0.001) | 0.005  (0.001) | 0.002  (0.001) | 0.005*  (0.002) | 0.016* (0.009) | 0.01* (0.003) |
|  | cy19.0 | **0.009***  **(0.003)** | **0.008**  **(0.002)** | 0.002  (0.001) | 0.006*  (0.002) | 0.018* (0.011) | 0.007 (0.003) |
| Gram positive bacteria | a15.0 | 0.004*  (0.001) | 0.004*  (0.001) | 0.003  (0.001) | 0.004*  (0) | 0.031* (0.017) | 0.07  (0) |
|  | a17.0 | 0.007*  (0.002) | 0.004  (0.001) | 0.004  (0.001) | 0.004*  (0.001) | 0.026* (0.014) | 0.022* (0.01) |
|  | i15.0 | 0.009*  (0.002) | 0.01  (0.002) | 0.007  (0.002) | 0.005*  (0.001) | 0.018* (0.012) | 0.015 (0) |
|  | i16.0 | 0.012*  (0.002) | 0.017*  (0.003) | 0.006  (0.002) | 0.009*  (0.001) | 0.031* (0.016) | 0.027* (0) |
|  | i17.0 | 0.01  (0.003) | 0.006  (0.003) | 0.007  (0.002) | 0.007*  (0.001) | 0.028* (0.017) | 0.021* (0.009) |
| Actinobacteria | 10Me17.0 | 0.003*  (0.001) | 0.001  (0) | 0.002  (0.001) | 0.003  (0.001) | 0.019* (0.013) | 0.011 (0.004) |
| General PLFAs | 16.0 | 0.175*  (0.026) | 0.206*  (0.041) | 0.062  (0.015) | 0.095  (0.041) | 0.046* (0.017) | 0.025* (0.008) |
|  | 18.0 | 0.065*  (0.009) | 0.073*  (0.017) | 0.027  (0.007) | 0.029  (0.01) | **0.037* (0.011)** | **0.016* (0.006)** |

**Table S3** Abundance of PLFAs, in µg C in PLFAs g^-1^ dry weight. Values are means, standard error is shown in brackets (n=6). Asterisks indicate significant difference to control (p < 0.05; Mann-Whitney U test). Bold text indicates significant difference between untreated (no N) and N-treated (+N) side (p < 0.05; Mann-Whitney U test for paired samples).

|  |  | **Rhizosphere soil** | | **Bulk soil** | | **Litter** | |
| --- | --- | --- | --- | --- | --- | --- | --- |
| Phylogenetic specificity | PLFA | no N | +N | no N | +N | no N | +N |
| Fungi | 18.1ω9cis | 11.207 (0.595) | 11.872  (0.439) | 10.68 (1.098) | 9.792  (1.02) | **34.218 (4.271)** | **20.511 (4.355)** |
|  | 18.1ω9trans | 5.73 (0.186) | 6.097  (0.338) | 5.833 (0.361) | 5.28  (0.266) | 25.705 (1.544) | 26.379 (4.648) |
|  | 18.2w6.9 | 3.63 (0.574) | 3.637  (0.623) | 2.621 (0.447) | 2.54  (0.291) | 21.375 (2.685) | 20.543 (2.962) |
| Bacteria general | 17.0 | **0.788 (0.098)** | **0.453**  **(0.081)** | 0.673 (0.089) | 0.554  (0.056) | 3.528 (0.585) | 1.546* (0.312) |
| Gram negative bacteria | 16.1ω5 | **1.647 (0.127)** | **1.177**  **(0.126)** | **1.649 (0.219)** | **0.948***  **(0.131)** | **5.271 (1.54)** | **1.17 (0.403)** |
|  | 16.1ω7 | **4.882 (0.321)** | **3.633**  **(0.32)** | **4.545 (0.591)** | **2.947***  **(0.372)** | **13.824 (3.051)** | **5.269* (0.473)** |
|  | cy17.0 | 2.271 (0.099) | 2.049  (0.103) | **2.234 (0.152)** | **1.742***  **(0.125)** | 9.295 (0.693) | 6.208* (0.232) |
|  | cy19.0 | 8.358* (0.822) | 10.342*  (0.669) | 8.032 (1.209) | 8.866  (1.372) | 14.479 (4.721) | 16.642 (7.27) |
| Gram positive bacteria | a15.0 | **0.937 (0.15)** | **0.449**  **(0.118)** | **1.229 (0.25)** | **0.28***  **(0.039)** | **2.513 (0.549)** | **0.018* (0.018)** |
|  | a17.0 | **1.942 (0.156)** | **1.251**  **(0.168)** | 1.746 (0.254) | 1.257  (0.144) | **5.956 (1.088)** | **2.789* (0.148)** |
|  | i15.0 | 0.969 (0.155) | 0.495  (0.133) | **1.237 (0.192)** | **0.329***  **(0.041)** | **3.73 (0.677)** | **0.173* (0.077)** |
|  | i16.0 | **1.286 (0.088)** | **0.907**  **(0.074)** | **1.473 (0.148)** | **0.727***  **(0.035)** | **6.046 (0.635)** | **1.853* (0.397)** |
|  | i17.0 | **1.987 (0.143)** | **1.466**  **(0.136)** | **1.802 (0.255)** | **1.261***  **(0.152)** | **5.586 (1.419)** | **2.221* (0.17)** |
| Actinobacteria | 10Me17.0 | **2.521 (0.195)** | **1.835**  **(0.185)** | **2.242 (0.358)** | **1.629***  **(0.245)** | **5.476 (1.744)** | **2.126 (0.343)** |
| General PLFAs | 16.0 | **5.997 (0.232)** | **4.607**  **(0.231)** | **5.906 (0.307)** | **3.609***  **(0.106)** | **28.075* (2.417)** | **11.945* (0.925)** |
|  | 18.0 | 2.986 (0.291) | 2.411*  (0.062) | **2.313 (0.189)** | **1.907**  **(0.145)** | **8.677 (0.844)** | **5.858* (0.35)** |

**Table S4** ^13^C enrichment in PLFAs, in ng ^13^C in PLFAs g^-1^ dry weight. Values are means, standard error is shown in brackets (n=6). Bold text indicates significant difference between untreated (no N) and N-treated (+N) side (p < 0.05; Mann-Whitney U test for paired samples).

|  |  | **Rhizosphere soil** | | **Bulk soil** | | **Litter** | |
| --- | --- | --- | --- | --- | --- | --- | --- |
| Phylogenetic specificity | PLFA | no N | +N | no N | +N | no N | +N |
| Fungi | 18.1ω9cis | 5.486 (0.716) | 4.56  (0.895) | 2.592 (0.632) | 1.888  (0.515) | **22.256 (9.242)** | **6.184 (2.13)** |
|  | 18.1ω9trans | 7.135 (1.31) | 6.718  (1.145) | 2.731 (0.638) | 3.32  (1.595) | **6.863 (2.087)** | **3.987 (1.653)** |
|  | 18.2ω6.9 | 24.492 (4.423) | 29.563  (5.919) | 8.394 (2.238) | 11.586  (5.487) | 17.288 (7.119) | 10.221 (5.276) |
| Bacteria general | 17.0 | 0.179 (0.032) | 0.108  (0.03) | 0.082 (0.024) | 0.056  (0.014) | **0.707 (0.22)** | **0.269 (0.163)** |
| Gram negative bacteria | 16.1ω5 | **0.168 (0.038)** | **0.05**  **(0.028)** | 0.075 (0.03) | 0.059  (0.013) | **0.539 (0.177)** | **0.076 (0.023)** |
|  | 16.1ω7 | **0.85 (0.207)** | **0.399**  **(0.118)** | 0.38 (0.142) | 0.207  (0.072) | **3.245 (1.242)** | **0.554 (0.163)** |
|  | cy17.0 | 0.12 (0.018) | 0.1  (0.023) | 0.057 (0.016) | 0.098  (0.039) | **1.222 (0.558)** | **0.612 (0.199)** |
|  | cy19.0 | 0.694 (0.19) | 0.806  (0.219) | **0.161 (0.056)** | **0.589**  **(0.251)** | 1.763 (0.874) | 0.73 (0.32) |
| Gram positive bacteria | a15.0 | **0.034 (0.007)** | **0.02**  **(0.008)** | 0.041 (0.013) | 0.01  (0.002) | **0.411 (0.089)** | **0.013 (0.013)** |
|  | a17.0 | 0.128 (0.025) | 0.042  (0.015) | 0.077 (0.031) | 0.053  (0.011) | 1.104 (0.417) | 0.613 (0.304) |
|  | i15.0 | **0.083 (0.016)** | **0.045**  **(0.017)** | 0.09 (0.025) | 0.017  (0.006) | **0.339 (0.091)** | **0.026 (0.013)** |
|  | i16.0 | 0.144 (0.024) | 0.148  (0.027) | 0.095 (0.028) | 0.066  (0.008) | **1.426 (0.453)** | **0.562 (0.334)** |
|  | i17.0 | 0.206 (0.052) | 0.084  (0.04) | 0.137 (0.049) | 0.094  (0.015) | **0.885 (0.278)** | **0.43 (0.141)** |
| Actinobacteria | 10Me17.0 | 0.087 (0.019) | 0.019  (0.006) | 0.045 (0.017) | 0.032  (0.008) | 0.498 (0.219) | 0.191 (0.056) |
| General PLFAs | 16.0 | 10.23 (1.315) | 9.402  (2.009) | 3.816 (0.938) | 3.448  (1.418) | 11.374 (3.7) | 2.91 (1.033) |
|  | 18.0 | 1.86 (0.191) | 1.775  (0.421) | 0.669 (0.163) | 0.62  (0.228) | **2.854 (0.635)** | **0.909 (0.308)** |
