# Supplementary material for: Rapid Transfer of Plant Photosynthates to Soil Bacteria via Ectomycorrhizal Hyphae and Its Interaction With Nitrogen Availability
Source: Front Microbiol. 2019 Feb 26;10:168. doi: 10.3389/fmicb.2019.00168 (PMC6399413; doi:10.3389/fmicb.2019.00168)
Supplement: Supplementary file 3 [file Data_Sheet_1.PDF]

Supplementary Figures

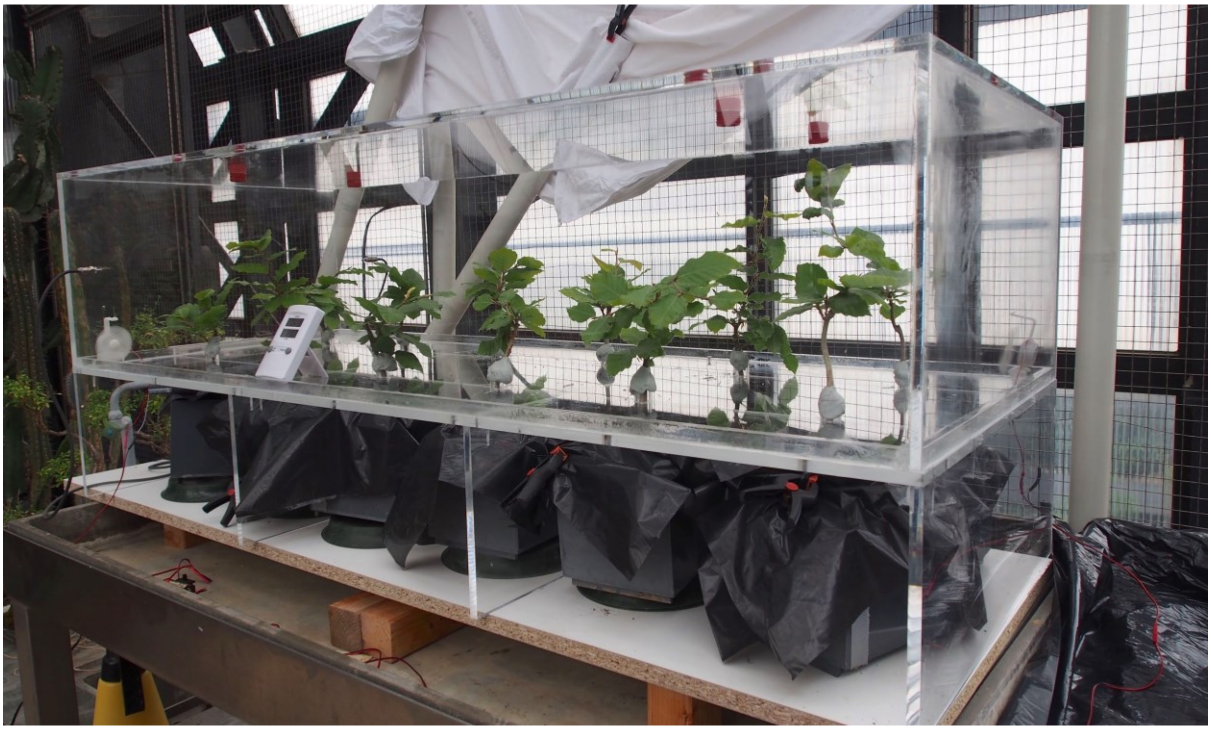

Figure S1 Labeling the plant in the labelling chamber. Plant canopies were exclusively labelled with ^{13}C-CO_2, sealed off with Therostat® adhesive (gray in color). An infrared gas analyzer was connected to the chamber. Two fans inside the chamber ensured a homogeneous atmosphere. ^{13}C-CO_2 was injected via red septa at the top of the chamber. Black plastic bags covered the split-root boxes to prevent photosynthesis.

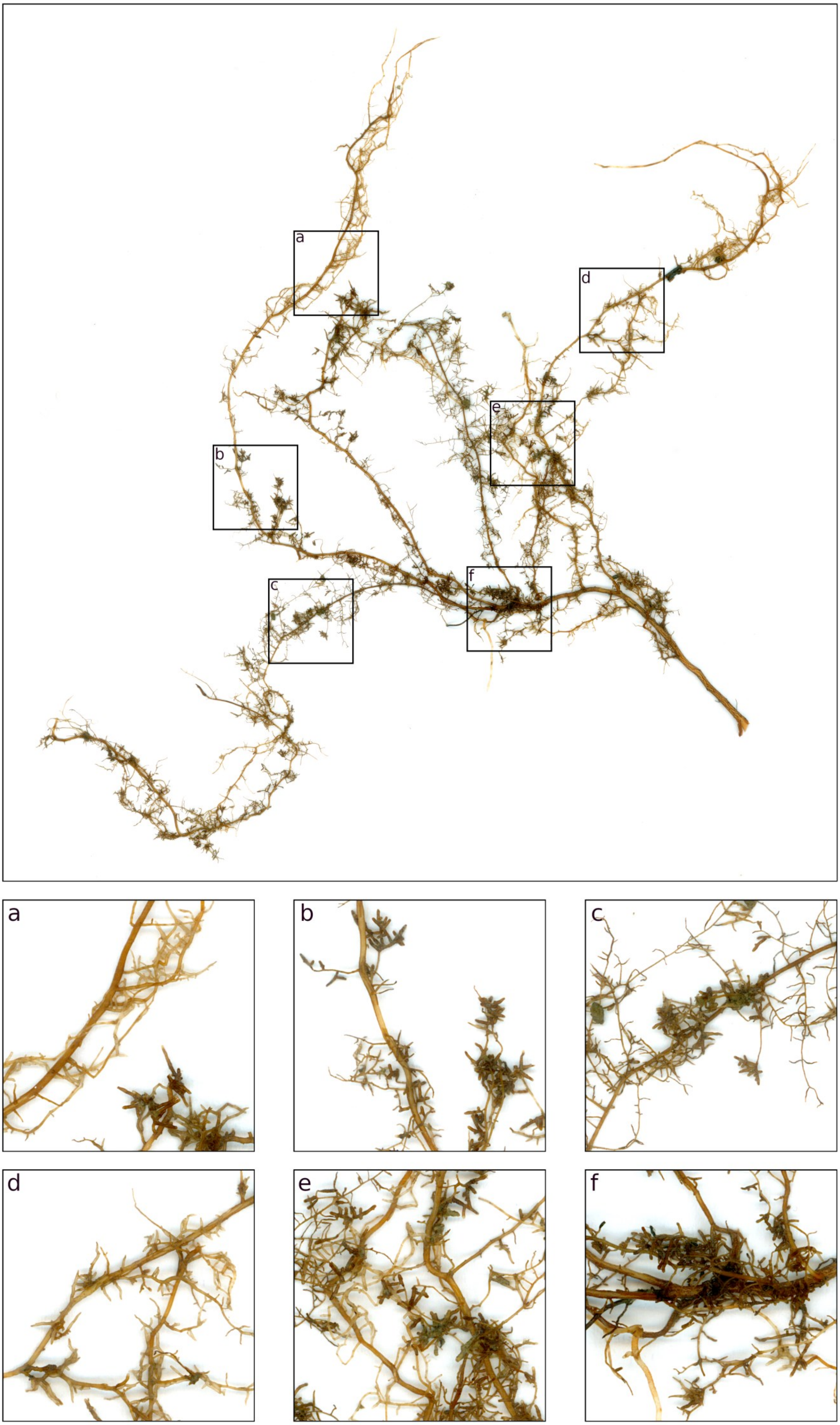
a
b
c
d
e
f
a
b
c
d
e
f

Figure S2 Mycorrhizal colonization on a typical root from an experimental plant. This scan was taken from part of the root system of the N-treated side of a ^{13}C and ^{15}N labeled plant, and is representative for all analyzed roots. Top picture, root scan overview; bottom pictures, enlarged regions showing mycorrhizal root tips.

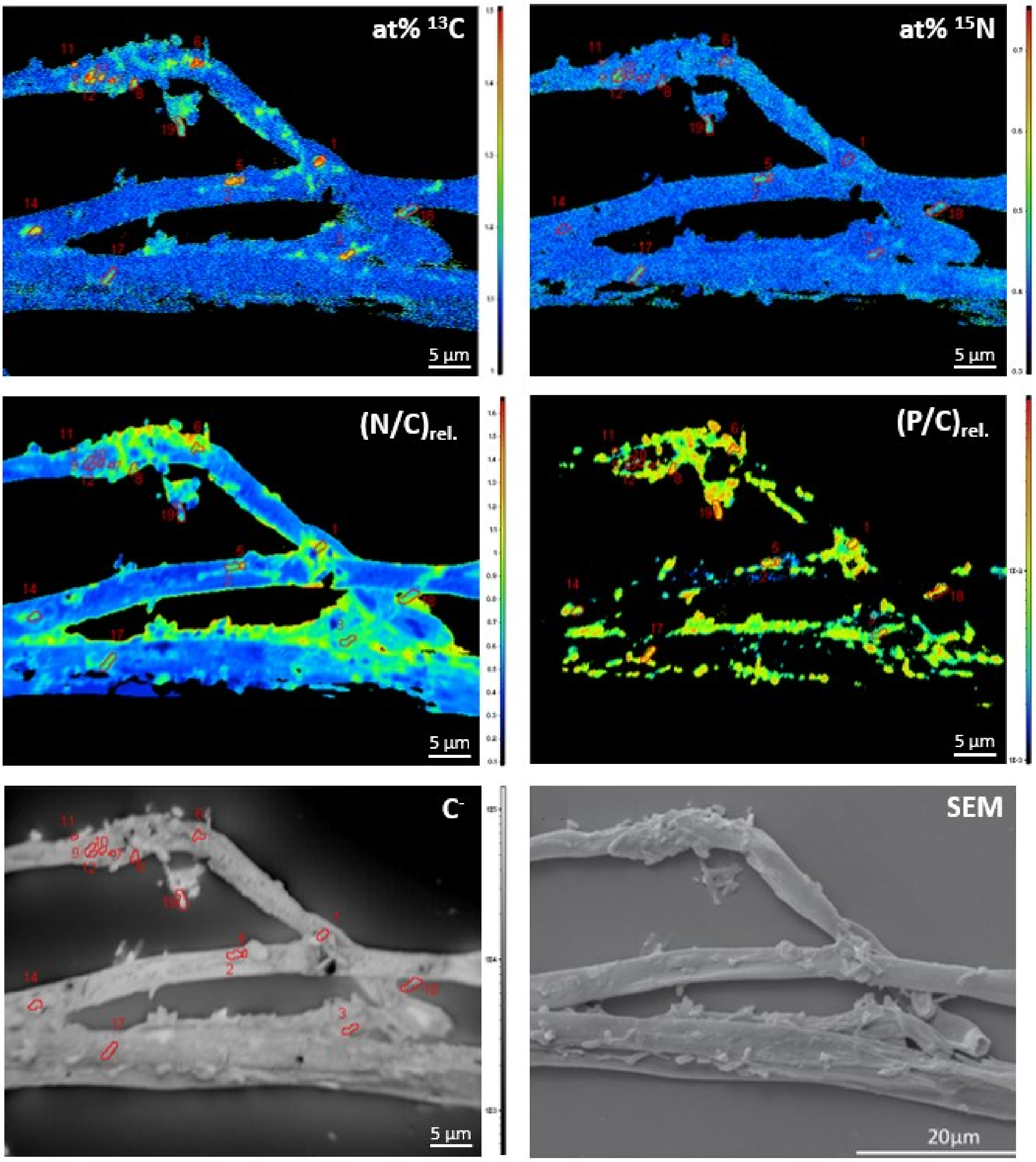

Figure S3 Regions of interest (ROI) for determination of the carbon and nitrogen isotope compositions of single microbial cells on hyphal surfaces. at% ^{13}C, carbon isotope composition image in atom% ^{13}C; at% ^{15}N, nitrogen isotope composition image in atom% ^{15}N; $(N/C)_{rel.}$, relative nitrogen-to-carbon elemental ratio image as inferred from C_2^- normalized CN^- secondary ion signal intensities, given in arbitrary units; $(P/C)_{rel.}$, relative phosphor-to-carbon elemental ratio image as inferred from C^- normalized P^- secondary ion signal intensities, given in arbitrary units; C^-, accumulated $^{12}C^-$ and $^{13}C^-$ secondary ion signal intensity distribution images, indicating the morphology of the sample during NanoSIMS analysis; SEM, scanning electron microscopy image (secondary electrons).

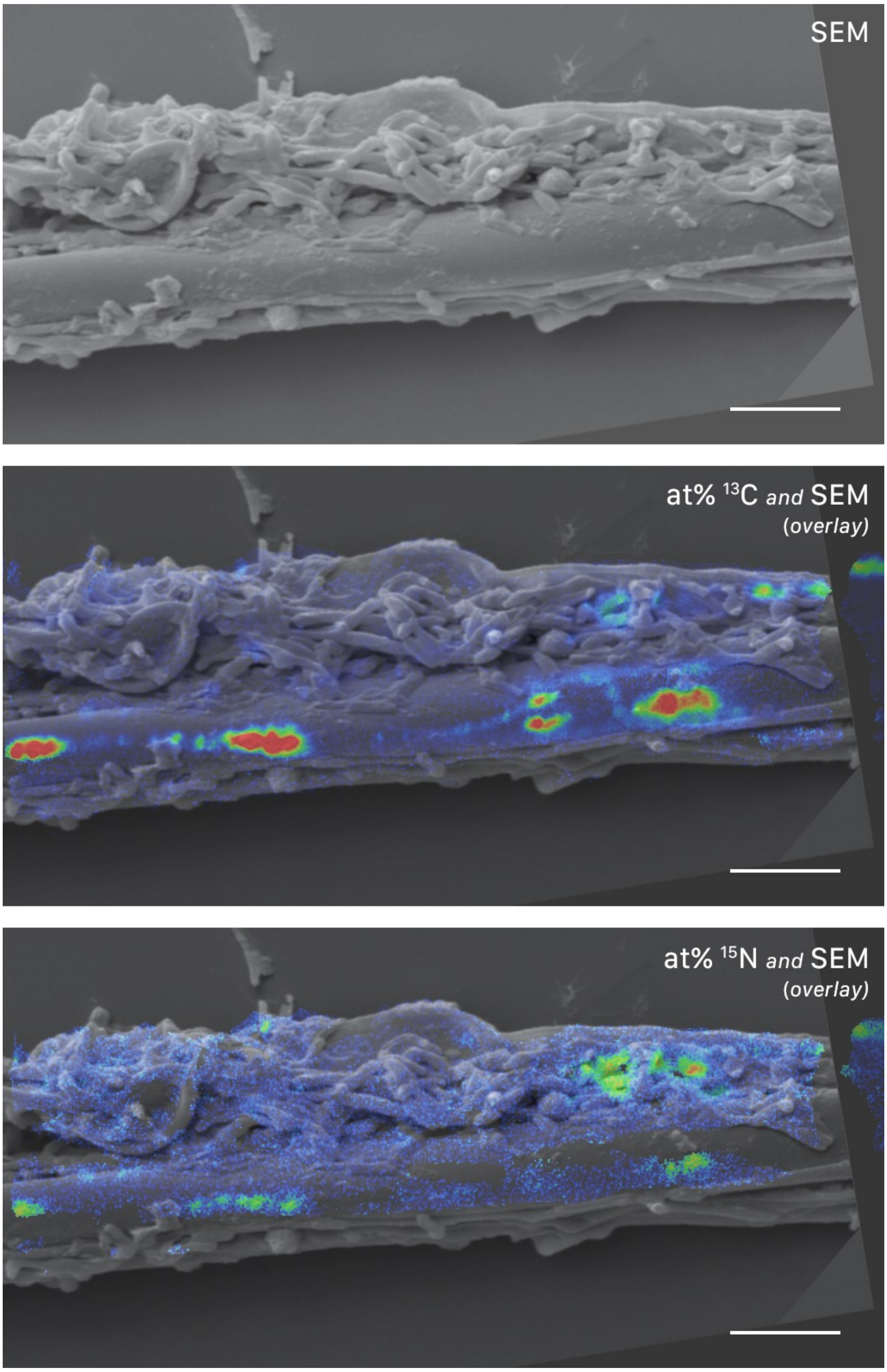

Figure S4 Addendum to **Figure 4**: Overlay of the isotope composition images (acquired in the lumen of the hypha) with the SEM image (recorded prior to NanoSIMS analysis) reveals that regions of high isotope enrichment were mainly located beneath the part of the hyphal surface devoid of microbial single cells. Scale bars, 5 µm.

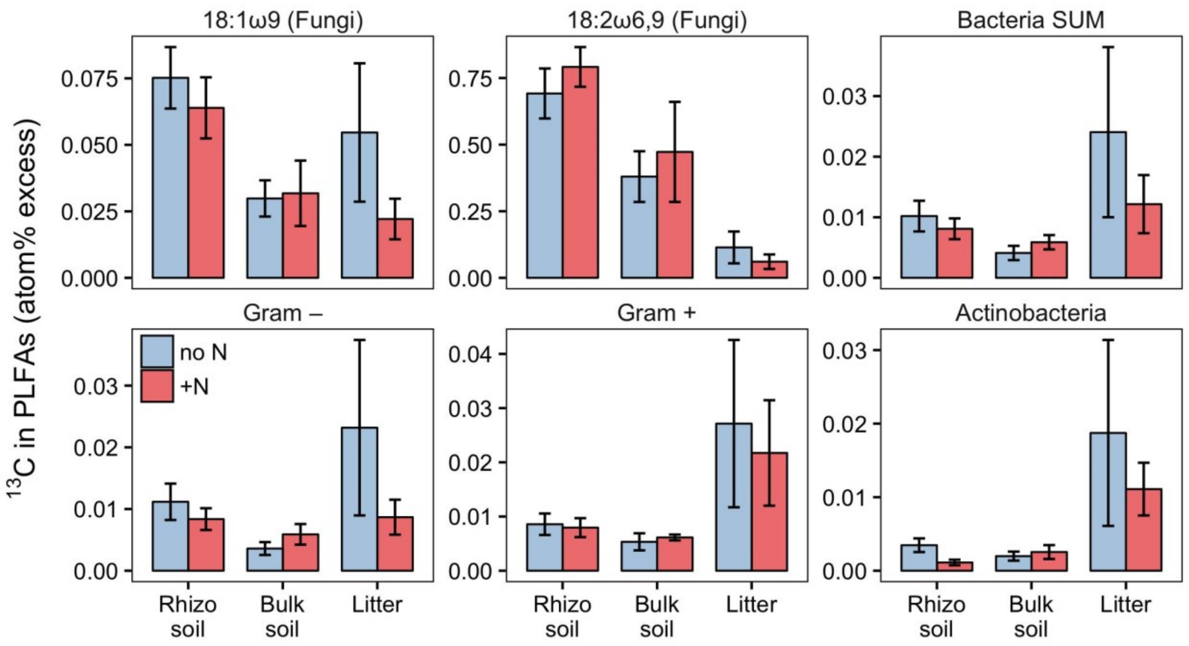

Figure S5 Enrichment of ^{13}C in fungal-specific (18:1ω9 and 18:2ω6,9) and bacteria-specific PLFAs in soil (rhizosphere soil, bulk soil) and litter compartments of the untreated (no N) and N-treated (+N) split-root box sides (cf. **Figure 3**, where the weighted mean of both untreated and N-treated side is shown). No significant differences between untreated and N-treated side could be detected (Mann-Whitney U test for paired samples, $p < 0.05$). Error bars represent the standard error.

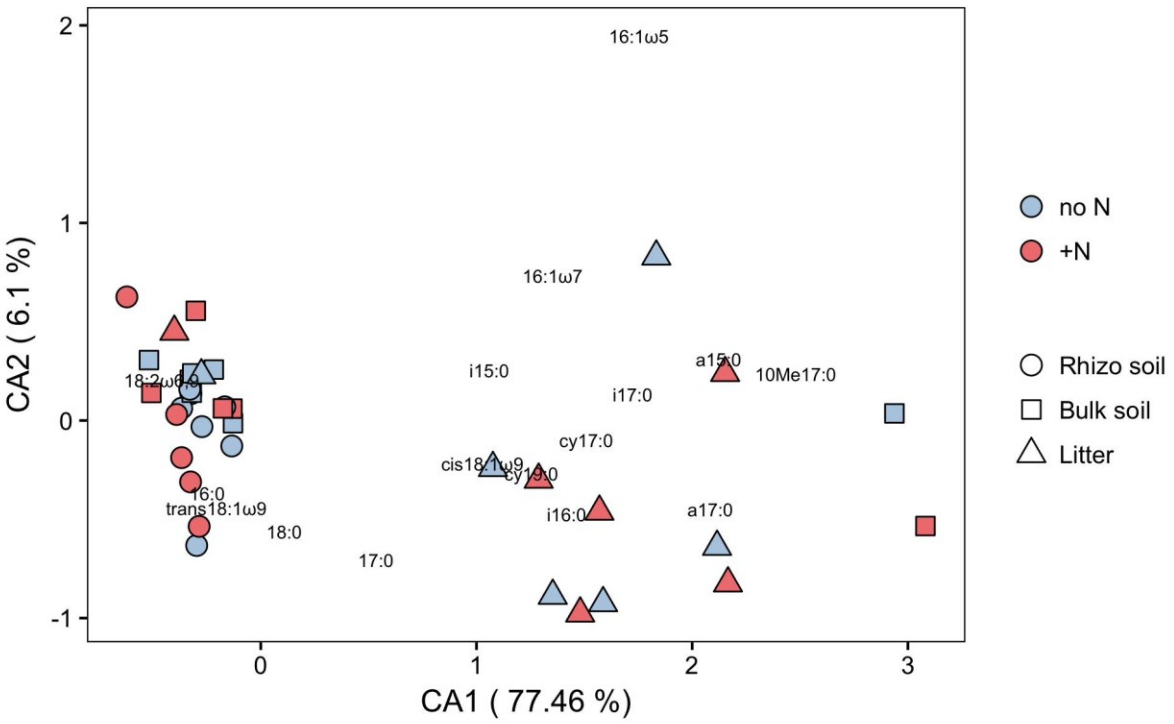

Figure S6 Correspondence analysis (CA) of atom% excess ^{13}C in PLFAs. Close distances between individual PLFAs (depicted as text) and soil/litter-pools of individual split-root boxes (depicted as symbols) indicate higher ^{13}C-enrichment of respective PLFAs in concerned pools. Axes notations give the proportion of variance explained on each ordinate in percent. Colors refer to box-sides with untreated (no N) and N-treated (+N) litter compartments. ANOSIM analysis shows significant differences between pools (R=0.396, p=0.001), and no difference between N-treatments (R=-0.022, p=0.755). This analysis indicates that ^{13}C was allocated to different microbes in soil (rhizosphere soil, bulk soil) and litter pools, respectively, but there is apparent difference in relative ^{13}C-enrichment between untreated and N-treated sides. Data points that fall out of line all belong to the same plant box.
