# Supplementary material for: Rapid Transfer of Plant Photosynthates to Soil Bacteria via Ectomycorrhizal Hyphae and Its Interaction With Nitrogen Availability
Source: Front Microbiol. 2019 Feb 26;10:168. doi: 10.3389/fmicb.2019.00168 (PMC6399413; doi:10.3389/fmicb.2019.00168)
Supplement: Supplementary file 4 [file Data_Sheet_2.pdf]

Supplementary methods

NanoSIMS

NanoSIMS measurements were performed on a NanoSIMS 50L (Cameca, Gennevilliers, France) at the Large-Instrument Facility for Advanced Isotope Research of the University of Vienna. Prior to data acquisition, analysis areas were pre-sputtered utilizing a high-intensity, slightly defocused Cs^+ ion beam (100 pA beam current, approx. 1 µm spot size). In order to avoid crater edge effects, scanning during pre-sputtering was conducted over areas with an edge length exceeding the frame size of the subsequently recorded images by at least 15 µm. For sampling of surface near regions on the fungal hyphae, pre-sputtering was finished after irradiation with Cs^+ ions to a fluence of 5.8E16 ions/cm^2, ensuring that analyzed regions were located within the bacterial cells. Analysis areas within the lumen of fungal hyphae were accessed by extended pre-sputtering to Cs^+ fluences up to 2.9E17 ions/cm^2 (e.g. **Figure 5**). Data were acquired as multilayer image stacks by sequential scanning of a finely focused Cs^+ primary ion beam (ca. 80 nm probe size at 2 pA beam current) over areas between 40 × 40 and 52 × 52 µm^2 at 512 × 512 pixel image resolution and a primary ion beam dwell time of 15 to 20 msec/(pixel*cycle). For enhancement of the measurement efficiency, imaging of individual hyphae with horizontal alignment was performed under vertical confinement of the scanning area. $^{12}C^-$, $^{13}C^-$, $^{12}C^{12}C^-$, $^{12}C^{13}C^-$, $^{12}C^{14}N^-$, $^{12}C^{15}N^-$, $^{31}P^-$ secondary ions as well as secondary electrons were detected simultaneously. The mass spectrometer was tuned for achieving a mass resolving power (MRP) of > 9.500 (according to Cameca's definition) for detection of C_2^- and CN^- secondary ions, accomplished through insertion of spectrometer entrance slit ES#4 and aperture slit AS#3 into the secondary ion beam path.

Images based on NanoSIMS measurement data were generated using the Open MIMS plugin (Poczatek *et al.*, 2009) in the FIJI package based on ImageJ (Schindelin *et al.*, 2012). Data pre-processing comprised corrections for positional variations, originating from primary ion beam and/or sample stage drift, detector dead-time (44 nsec) and quasi-simultaneous arrival (QSA) of secondary ions. The applied QSA sensitivity factors ('beta-values'), obtained from measurements on dried yeast cells (data not shown), were 1.1, 1.06 and 1.05 for C^-, C_2^- and CN^- secondary ions, respectively. Carbon isotope composition images displaying the $^{13}C/(^{12}C+^{13}C)$ isotope fraction, designated as atom% ^{13}C, were inferred from the C^- and C_2^- secondary ion signal intensity distribution images via per-pixel calculation of $^{13}C^-/(^{12}C^-+^{13}C^-)$ and $^{13}C^{12}C^-/(2\cdot{}^{12}C^{12}C^-+^{13}C^{12}C^-)$ intensity ratios. Owing to superior counting statistics, all C isotope composition data shown in the manuscript were inferred from C_2^- signal intensities. We note that, in consistency with a recent study (Zumstein *et al.*, 2018), we did not observe any significant differences between ^{13}C content values inferred from C_2^- signal intensities versus C^- signal intensities. Nitrogen isotope composition images displaying the $^{15}N/(^{14}N+^{15}N)$ isotope fraction, designated as atom% ^{15}N, were inferred from the $^{12}CN^-$ secondary ion signal intensity maps via per-pixel calculation of $^{12}C^{15}N^-/(^{12}C^{15}N^-+^{12}C^{14}N^-)$ intensity ratios.

For visualization of relative phosphor and nitrogen elemental distributions, carbon (i.e. matrix) associated secondary ion signals were utilized as reference signals. As such, relative phosphor-to-carbon ratios, designated as $(P/C)_{rel.}$ given in arbitrary units [a.u.], were inferred from C^- normalized $^{31}P^-$ signal intensities via $^{31}P^-/(^{12}C^-+^{13}C^-)$. Correspondingly, relative nitrogen-to-carbon elemental ratios, designated as $(N/C)_{rel.}$ given in arbitrary units [a.u.], were obtained from C_2^- normalized CN^- signal intensities via $[^{12}C^{14}N^-(1+R_{13C/12C}) + ^{12}C^{15}N^-(1+R_{13C/12C})] / [^{12}C^{13}C^- + ^{12}C_2^-(1+R_{13C/12C}{}^2)]$, whereby the term $R_{13C/12C}$ refers to the ^{13}C-to-^{12}C isotope ratio, calculated from the C_2^- signal intensities via $^{13}C^{12}C^- /(2\cdot{}^{12}C^{12}C^-)$. It should be noted that we used atomic ions (C^-) as reference signals for atomic (P^-) secondary ions, whereas molecular ions (C_2^-) were used as reference signals for molecular (CN^-) ions in order to minimize potential biasing brought about by sample topography (Thomen *et al*., 2014).

Overlay images, combining morphological with chemical information, were assembled using GIMP 2.10.4 (GNU Image Manipulation Program, https://www.gimp.org/). SEM (secondary electron) images where utilized for representation of the sample morphology prior to NanoSIMS analysis. For visualization of the sample morphology evolving during NanoSIMS imaging, C^- signal intensity distribution images were used. In the context of angular aberration, it is worthy to mention that, according to the larger width of the slits and the less stringent conditions for beam focusing in the vertical plane of the mass spectrometer, the filamentous samples were preferentially aligned parallel to the horizontal plane of the spectrometer (see comments with respect to sample mounting given above). **Animation S1**, illustrating the variation of morphology and isotope distribution patterns within the measurement in the luminal sections of a fungal hypha (**Figure 5**), was assembled with the FIJI package based on ImageJ (Schindelin *et al.*, 2012). Details about the parameters applied for composition of the individual images of the z-stack are provided in the legend which is embedded in the animation.

Region of interest (ROI) specific numerical data evaluation was conducted using the WinImage software package (version 2.0.8) provided by Cameca. ROIs referring to individual microbial cells (**Figure 7**) were manually defined based on the relative N/C and P/C elemental ratio maps (serving as indicators for biomass) and cross-checked by the morphological appearance in the C^- secondary ion intensity distribution and SEM images (**Figure S3).** Within the fungal lumen (**Figure 5**), ROIs were defined according to the isotope enrichment patterns since it was not possible to identify characteristic cellular features. This may originate from accessing the luminal regions via depth-profiling, which is less suited for visualization of cellular (ultra)structure than e.g. cross-sectional analysis of resin embedded samples. C and N isotope compositions were calculated from the accumulated intensities of $^{12}C^{12}C^-$ and $^{12}C^{13}C^-$ and $^{12}C^{14}N^-$ and $^{12}C^{15}N^-$ secondary ion signals detected within each ROI. The isotopic composition values summarized in the boxplot shown in **Figure 6** were determined by averaging over the individual images of the multilayer stack (ranging from 16 to 24 individual cycles). As such, the displayed values may rather be considered as conservative estimates for the isotopic enrichment due to carry-over (e.g.

through re-deposition and/or atomic mixing) of atoms and molecules from less enriched regions within the fungal hyphae during the measurement process (including pre-sputtering).

The analytical uncertainty of the isotope composition values (indicated by the error bars in **Figure 6**), emerging from the random error in single ion counting, was estimated on the basis of Poisson statistics and calculated from the signal intensities (given in total counts within individual ROIs) via:

atom% ^{13}C inferred from C^- signal intensities:

$\sigma_{Pois}=1/(^{12}C^-+^{13}C^-)^2 \cdot sqrt((^{12}C^-)^2 \cdot ^{13}C^-+(^{13}C^-)^2 \cdot ^{12}C^-)$

atom% ^{13}C inferred from C_2^- signal intensities:

$\sigma_{Pois}=1/(2 \cdot ^{12}C^{12}C^-+^{12}C^{13}C^-)^2 \cdot sqrt((^{12}C^{12}C^-)^2 \cdot ^{12}C^{13}C^-+(^{12}C^{13}C^-)^2 \cdot ^{12}C^{12}C^-)$

atom% ^{15}N inferred from CN^- signal intensities:

$\sigma_{Pois}=1/(^{12}C^{14}N^-+^{12}C^{15}N^-)^2 \cdot sqrt((^{12}C^{14}N^-)^2 \cdot ^{12}C^{15}N^-+(^{12}C^{15}N^-)^2 \cdot ^{12}C^{14}N^-)$

References

Poczatek, C., Kaufman, Z., and Lechene, C. (2009). OpenMIMS ImageJ Plugin Guide. *Harvard Medical School (Boston, Massachusetts, USA).*

Schindelin, J., Arganda-Carreras, I., Frise, E., Kaynig, V., Longair, M., Pietzsch, T., et al. (2012). Fiji: an open-source platform for biological-image analysis. *Nature Methods* 9, 676–682. doi:10.1038/nmeth.2019.

Thomen, A., Robert, F., and Remusat, L. (2014). Determination of the nitrogen abundance in organic materials by NanoSIMS quantitative imaging. *Journal of Analytical Atomic Spectrometry* 29, 512. doi:10.1039/c3ja50313e.

Zumstein, M. T., Schintlmeister, A., Nelson, T. F., Baumgartner, R., Woebken, D., Wagner, M., et al. (2018). Biodegradation of synthetic polymers in soils: Tracking carbon into CO2 and microbial biomass. *Science Advances* 4, eaas9024. doi:10.1126/sciadv.aas9024.
