# Supplementary figures and images for: Rapid Transfer of Plant Photosynthates to Soil Bacteria via Ectomycorrhizal Hyphae and Its Interaction With Nitrogen Availability
Source: Front Microbiol. 2019 Feb 26;10:168. doi: 10.3389/fmicb.2019.00168 (PMC6399413; doi:10.3389/fmicb.2019.00168)

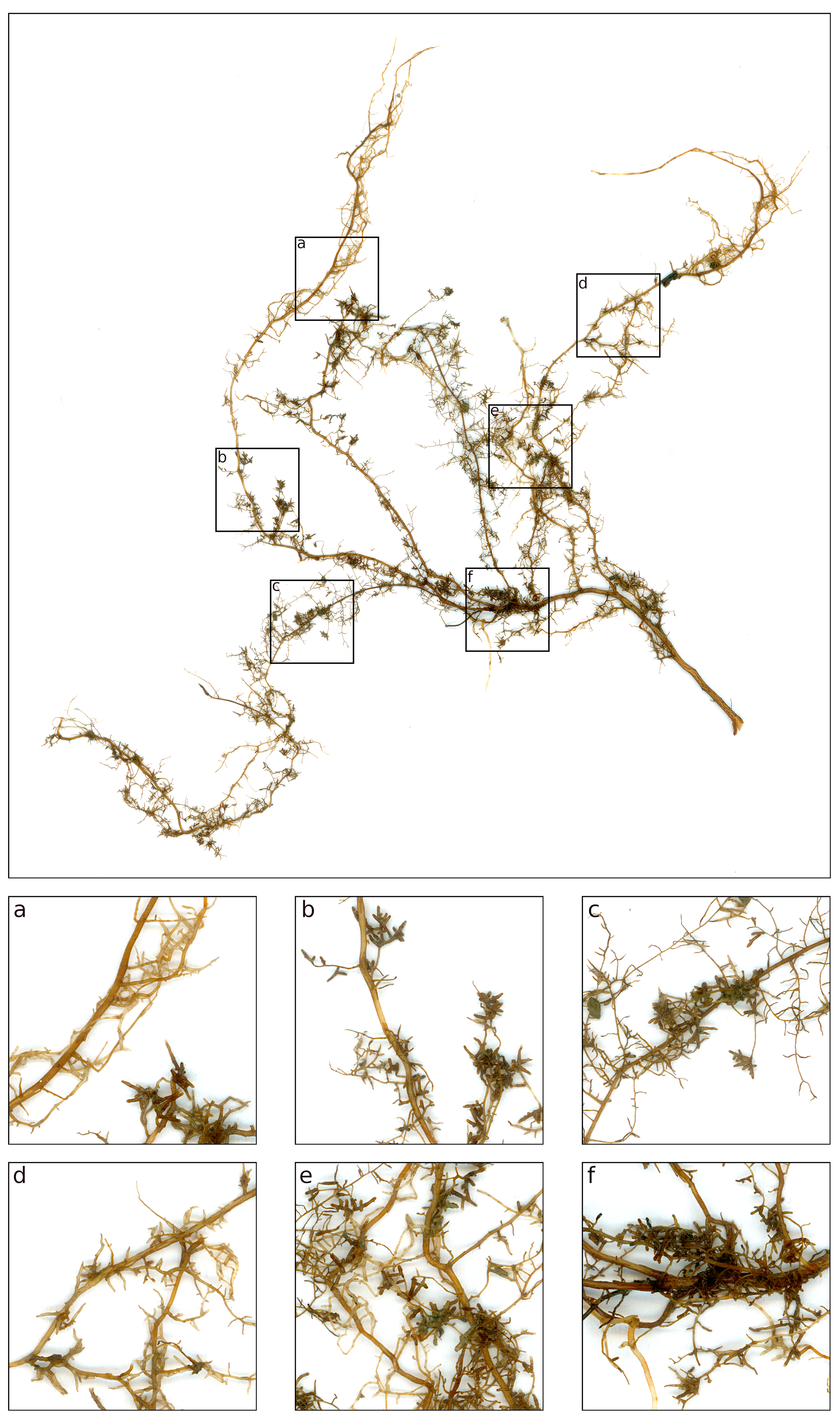

Supplement: Supplementary file 5 [file Image_1.PNG]
